# Innate, non-cytolytic CD8^+^ T cell-mediated suppression of HIV replication by MHC-independent inhibition of virus transcription

**DOI:** 10.1371/journal.ppat.1008821

**Published:** 2020-09-17

**Authors:** Michelle Zanoni, David Palesch, Claudia Pinacchio, Maura Statzu, Gregory K. Tharp, Mirko Paiardini, Ann Chahroudi, Steven E. Bosinger, Jack Yoon, Bryan Cox, Guido Silvestri, Deanna A. Kulpa

**Affiliations:** 1 Division of Microbiology and Immunology, Yerkes National Primate Research Center, and Emory Vaccine Center Emory University, Atlanta, Georgia, United States of America; 2 Department of Pediatrics, Emory University School of Medicine, Atlanta, Georgia, United States of America; 3 Department of Pathology and Laboratory Medicine, Emory University School of Medicine, Atlanta, Georgia, United States of America; Vaccine Research Center, UNITED STATES

## Abstract

MHC-I-restricted, virus-specific cytotoxic CD8^+^ T cells (CTLs) may control human immunodeficiency virus (HIV) and simian immunodeficiency virus (SIV) replication via the recognition and killing of productively infected CD4^+^ T cells. Several studies in SIV-infected macaques suggest that CD8^+^ T cells may also decrease virus production by suppressing viral transcription. Here, we show that non-HIV-specific, TCR-activated non-cytolytic CD8^+^ T cells suppress HIV transcription via a virus- and MHC-independent immunoregulatory mechanism that modulates CD4^+^ T cell proliferation and activation. We also demonstrate that this CD8^+^ T cell-mediated effect promotes the survival of infected CD4^+^ T cells harboring integrated, inducible virus. Finally, we used RNA sequencing and secretome analyses to identify candidate cellular pathways that are involved in the virus-silencing mediated by these CD8^+^ T cells. This study characterizes a previously undescribed mechanism of immune-mediated HIV silencing that may be involved in the establishment and maintenance of the reservoir under antiretroviral therapy and therefore represent a major obstacle to HIV eradication.

## Author summary

Antiretroviral therapy (ART) has revolutionized the fight against the HIV pandemic, but this approach requires life-long treatment in order to maintain suppression of virus replication, as rapid viral rebound follows ART discontinuation. Our current inability to cure HIV infection despite ART is due to a persistent reservoir of latently infected CD4^+^ T cells harboring replication competent HIV integrated in their genome. Advancing our understanding of how the virus reservoir is established and maintained in the setting of ART-treated HIV-infection may reveal novel targets for immune-based interventions that can eradicate HIV. In this study, we developed an innovative co-culture experimental system to identify and characterize an important antiviral and immunoregulatory activity of non-cytolytic CD8^+^ T lymphocytes on activated CD4^+^ T cells. This activity drives CD4^+^ T cells into a more resting state that is less conducive to HIV transcription, thereby promoting the survival of infected CD4^+^ T cells harboring integrated, inducible virus. In addition, we identified cellular pathways that may mediate the CD8^+^ T cell-mediated transcriptional silencing of HIV in CD4^+^ T cells. The possibility that this non-cytolytic activity of CD8^+^ T cells may sustain the *in vivo* pool of latently infected CD4^+^ T cells under ART therefore represents a key, previously unrecognized obstacle to the elimination of the virus reservoir and the eradication of HIV infection.

## Introduction

Several lines of experimental evidence suggest that CD8^+^ T cells play a significant role in the control of virus replication during the acute and chronic phases of HIV and SIV infection (reviewed in[1]). Correlative evidence includes the temporal association between the development of HIV/SIV-specific CTL responses and post-peak decline of viremia[2, 3], the emergence of virus CTL escape mutants[4, 5], the association between certain MHC class I haplotypes and disease progression[6, 7], and the presence of vigorous CTL responses in the so-called “elite controllers” (EC), persons living with HIV (PLWH) that show very low levels of viremia in absence of antiretroviral therapy (ART)[8].

The most direct evidence in favor of a major role of CD8^+^ T cells in controlling virus replication derives from studies in which the depletion of CD8^+^ T lymphocytes in SIV-infected macaques was consistently followed by increased virus replication[9–12]. However, the exact contribution of CTL activity to the suppression of HIV/SIV replication *in vivo* remains incompletely understood, and several studies suggest that non-cytolytic mechanisms of virus suppression (i.e., blockage of R5-tropic HIV *de novo* infection by beta-chemokines[13], and/or inhibition of virus transcription by unknown soluble factors[14, 15]) may also play an important, non-mutually exclusive role.

Intriguing evidence in support of the role of non-cytolytic mechanisms of virus suppression was first provided by two independent studies showing that CD8^+^ T lymphocyte depletion resulted in increased viremia in SIV-infected rhesus macaques (RMs) without prolonging the average *in vivo* lifespan of productively infected cells[16, 17]. The conclusion that non-cytolytic mechanisms of virus suppression are involved in controlling HIV/SIV replication is further supported by two additional independent studies.

In the first study, Al Basatena et al. used a 3D cellular automaton model of HIV infection that reproduces *in vivo* viral and cellular dynamics to demonstrate that non-cytolytic effector mechanisms can select for viral escape variants and provide more durable control of virus replication[18]. In the second study, Balamurali et al. used a RT-PCR assay that differentiates wild type (WT) virus from escape mutants (EM) to study the dynamics of immune escape in early simian/human immunodeficiency virus (SHIV) infection of pigtail macaques. These authors hypothesized that the death rate of WT infected cells (that are susceptible to CTL killing) would be faster than EM-infected cells, but found no significant difference in the rate of decay of WT versus EM virus, thus suggesting non-cytolytic control of both WT and EM variants of SHIV[19]. In more recent studies, we have shown that (i) CD8^+^ lymphocyte depletion is followed by significant reactivation of virus production in ART-treated, SIV-infected RMs[20], and that (ii) CD8^+^ lymphocyte depletion augments dramatically the effect of the latency-reversing agent (LRA) IL-15 superagonist N-803 in ART-treated, SIV-infected RMs, and in HIV-infected humanized mice [21]. Collectively, these studies indicate that CD8^+^ T cells can control HIV/SIV production via non-cytolytic mechanisms that may include transcriptional suppression of integrated proviruses.

Activated CD4^+^ T cells are highly susceptible to HIV infection both *in vitro* and *in vivo*, and the vast majority of the productively infected CD4^+^ T cells dies rapidly from (i) activation-induced cell death[22], (ii) viral cytopathic effects (CPE)[23, 24], or (iii) lysis by CTLs[4, 25, 26]. However, a small pool (~1 per 10^6^ cells[27]) of non-productively infected, resting CD4^+^ T cells which harbor replication-competent virus survive and transition to a long-lived, memory resting state[28]. This reservoir of latently-infected CD4^+^ T cells persists for years despite prolonged suppression of viral replication during ART[29, 30], thereby presenting a major barrier to cure HIV infection. It should be noted that, in the setting of full suppression of virus replication occurring *in vivo* under ART, we hypothesize that clearance of infected cells via CTL activity will result in a net decrease of the reservoir size, while the active suppression of HIV or SIV transcription in CD4^+^ T cells by non-cytolytic mechanisms of virus suppression may paradoxically favor the reservoir persistence by actively promoting latency.

Given that HIV-1-specific CTL responses have not been detected in CD8^+^ T cells isolated from HIV-negative subjects by either staining (pentamer, IFN-γ, and perforin) or ELISpot [31–33], in the current study we examined the distinct contribution of those CD8^+^ T cells to the suppression of viral transcription (we will further refer to these non-HIV-specific CD8^+^ T cells isolated from healthy HIV-negative subjects simply as CD8^+^ T cells, as opposed to the HIV-specific CD8^+^ T cells (CTLs) that are found in PLWH). To this end, we infected CD4^+^ T cells from HIV-uninfected subjects using different models of infection, and then co-cultured with autologous CD8^+^ T cells. We elected to limit HIV infection to one replication cycle, by either blocking the production of mature viral particles with the protease inhibitor Darunavir, or by infecting CD4^+^ T cells with a replication-incompetent virus. This experimental set-up allowed us to focus on CD8^+^ T cell-mediated antiviral effects that are related to post-entry processes, such as viral transcription.

We found that non-HIV-specific, TCR-activated CD8^+^ T cells potently suppress HIV transcription via a virus- and MHC-independent immunoregulatory mechanism that reduces CD4^+^ T cell activation and promotes the survival of infected CD4^+^ T cells harboring integrated, inducible virus. Finally, we used RNA sequencing and secretome analyses to identify candidate cellular signals that may govern this non-cytolytic CD8^+^ T cell-mediated suppression of HIV transcription in CD4^+^ T cells.

## Results

### TCR-stimulated CD8^+^ T cells effectively suppress HIV expression and replication in CD4^+^ T cells

**Fig 1:**
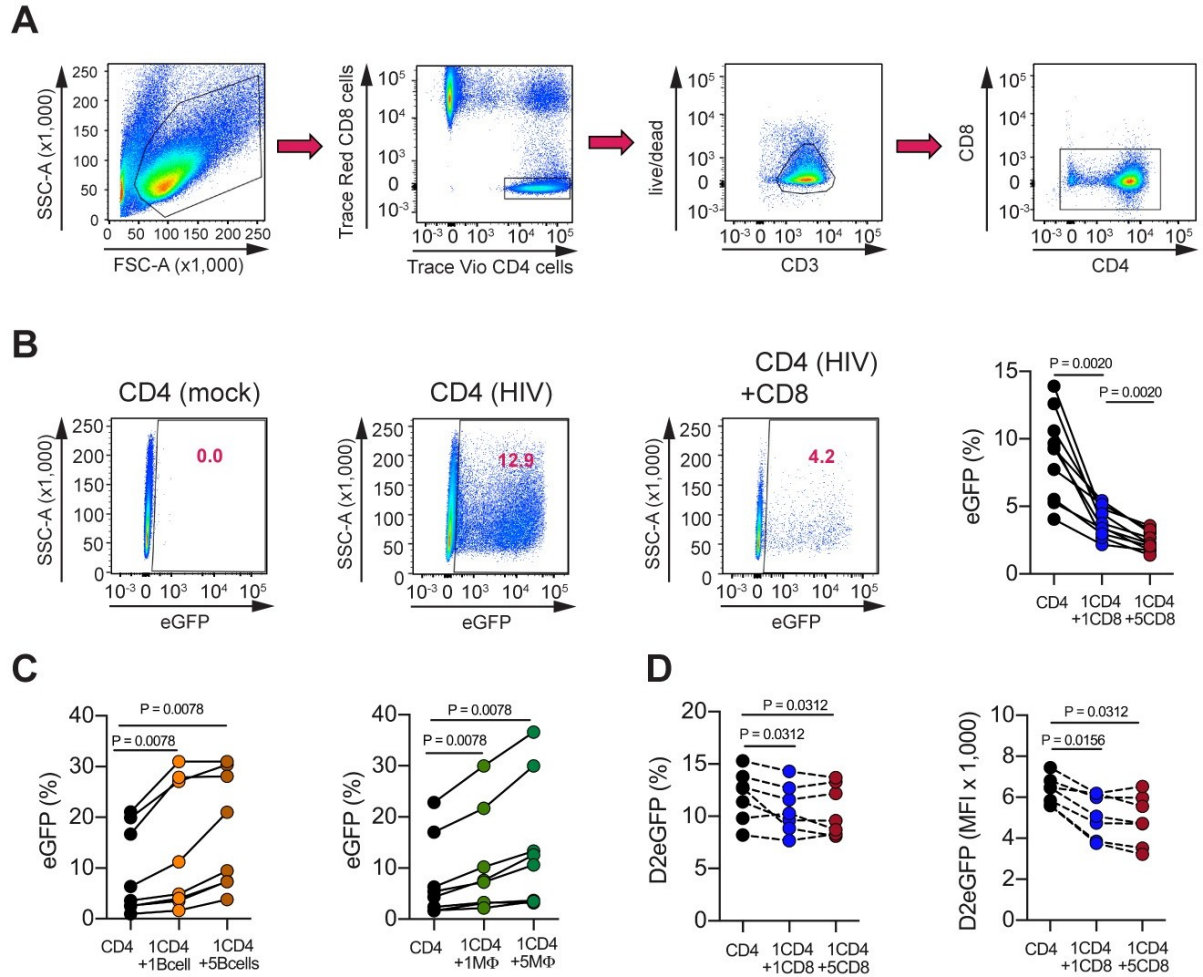
TCR-stimulated CD8^+^ T cells but not activated B cells or monocytes mediate suppression of HIV replication in CD4^+^ T cells. **(A**) CD4/CD8 Flow Cytometry Gating Strategy shows isolated CD4^+^ and CD8^+^ T cells separately labeled with CellTrace Violet dye (Vio^+^) and CellTrace Red dye (Red^+^). (**B**) Representative flow plots of uninfected (mock), and infected wells (HIV) depicting productively infected CD4^+^ T cells (live CD3^+^Vio^+^Red^-^CD8^-^eGFP^+^ cells) from CD4 mono- and CD4/CD8 co-cultures; the levels of suppression are represented by solid lines connecting the frequencies of eGFP^+^ cells from CD4 mono-culture wells (black), CD4/CD8 at 1:1 (blue) and 5:1 (red) E:T ratios from each subject (n = 10 subjects). (**C**) Co-culture with activated B cells or Monocytes. The frequencies of productively infected CD4^+^ T cells are shown from the CD4 mono-culture wells (black), CD4/CD19 at 1:1 (light orange) and 5:1 (dark orange) E:T ratios; CD4/CD14 at 1:1 (light green) and 5:1 (dark green) E:T ratios. eGFP^+^ levels are individual values from 8 HIV-negative distinct subjects with solid lines connecting values from mono- and co-cultures from each subject. (**D**) Infection with Env-defective NL4-3_D2eGFP virus. The levels of suppression are shown by dashed lines connecting the frequencies and mean fluorescence intensity (MFI) of D2eGFP^+^ cells from CD4 mono-culture wells (black), CD4/CD8 at 1:1 (blue) and 5:1 (red) E:T ratios from each subject (n = 7 subjects). Flow cytometric data for all the samples were performed on day 3 post- HIV infection, and comparisons between frequencies and MFI of HIV expression on mono- and co-cultures were carried out using Wilcoxon matched-pairs signed rank test.

We developed a dual-color CD4/CD8 co-culture assay to examine the effect of autologous TCR-activated CD8^+^ T cells from HIV-uninfected healthy subjects in modulating the *ex-vivo* infection of CD4^+^ T cells. To account for the HIV Nef-mediated downmodulation of CD4 in infected cells[34], we separately labelled CD4^+^ T cells and CD8^+^ T cells with CellTrace Violet dye (Vio) and CellTrace Red dye (Red), respectively, and prior to infection with a NL4-3 reporter virus expressing eGFP under control of the HIV- 1 LTR. This strategy allowed us to distinguish both T cell subsets following co-culture and to accurately analyze eGFP expression in infected CD4^+^ T cells regardless of the current level of CD4 surface expression. As such, in this assay we defined productively infected CD4^+^ T cells as live CD3^+^Vio^+^Red^-^CD8^-^eGFP^+^ cells, whereas the cell population that comprises non-productively infected cells as well as uninfected cells, was identified as live CD3^+^Vio^+^Red^-^CD8^-^eGFP^-^ (Fig 1A and 1B).

To test whether autologous CD8^+^ T cells from HIV-uninfected healthy subjects suppress viral replication in infected CD4^+^ T cells, TCR-activated Red^+^CD8^+^ T cells were added after infection of Vio^+^CD4^+^ T cells with a replication-competent NL4-3_eGFP virus, and then co-cultured for 72 hours at 1:1 and 5:1 CD8 to CD4 ratios. We then compared the frequencies of productively infected CD4^+^ T cells from CD4/CD8 co-cultures to the frequencies of productively infected CD4^+^ T cells from CD4 mono-cultures. We found that autologous TCR-activated CD8^+^ T cells from healthy subjects significantly suppressed HIV_eGFP expression in a dose-dependent manner with a mean reduction of 56% and 73% at 1:1 and 5:1 effector-to-target cell (E:T) ratios, respectively (p = 0.0020; Fig 1B).

Next, we addressed the possibility that the observed suppression of HIV infection was due to a non-specific, cell type-independent effect, e.g. a hindrance of cell-to-cell interaction in co-culture. To this end, autologous activated Red^+^CD19^+^ B cells or activated Red^+^CD14^+^ monocytes were added to the infected Vio^+^CD4^+^ T cells, and subjected to the same culture conditions as described above. In contrast to the effect observed with CD8^+^ T cells, B cells and monocytes failed to suppress HIV expression and exhibited even slightly higher frequencies of productively infected CD4^+^ T cells than observed in CD4^+^ T cell mono-cultures (Fig 1C), thus indicating a specific role of CD8^+^ T cells in suppressing HIV expression.

Using a replication-competent reporter virus in this experimental setup enables the infection of new target cells thus resulting in viral spread during the cellular culture. To determine whether the CD8^+^ T cell-mediated suppressive activity is affecting pre- or post-entry viral replication, we next used two separate models of single cycle of infection in our dual color CD4/CD8 assay: (i) by blocking the production of mature viral particles from the replication-competent NL4-3_eGFP virus with the protease inhibitor Darunavir; and (ii) by infecting CD4^+^ T cells with a replication-incompetent, Envelope (Env)-defective NL4-3_eGFP complemented in trans with a dual-tropic Env. For both single cycle settings, we did not observe significant changes in the levels of eGFP expression in the presence of CD8^+^ T cells compared to CD4^+^ T cell mono-cultures (S1A Fig). However, given that an enhanced half-life of 26 hours could explain the lack of suppression observed in the eGFP expression, and to gain a better understanding on the dynamic process that regulates gene expression, we next modified the replication-incompetent, Env-defective NL4-3_eGFP construct described above to express a short-lived, destabilized version of the eGFP reporter gene[35] under the control of the viral LTR (NL4-3_D2eGFP; S1B Fig). As expected, we then observed consistent levels of viral suppression in this single cycle setting, both by frequency of D2eGFP^+^ cells and mean fluorescence intensity (MFI), indicating that CD8^+^ T cells from HIV-naïve healthy subjects not only inhibit virus spread (Fig 1B), but also mediate a direct inhibitory effect on HIV LTR-driven expression on a per-infected-cell basis (Fig 1D).

### CD8^+^ T cell-mediated HIV suppression reflects a virus-independent mechanism of modulation of CD4^+^ T cell proliferation and activation

Numerous studies have shown that CD8^+^ T cells modulate CD4^+^ T cell activation and proliferation in the setting of autoimmunity [36, 37], transplant rejection[38, 39], and anti-tumor immune responses[40, 41]. Since HIV transcription is generally more effective in activated CD4^+^ T cells as compared to resting CD4^+^ T cells[42], we next sought to evaluate whether and to what extent the observed effect of CD8^+^ T cells on HIV replication was related to an effect on the activation and proliferation levels of infected CD4^+^ T cells.

**Fig 2:**
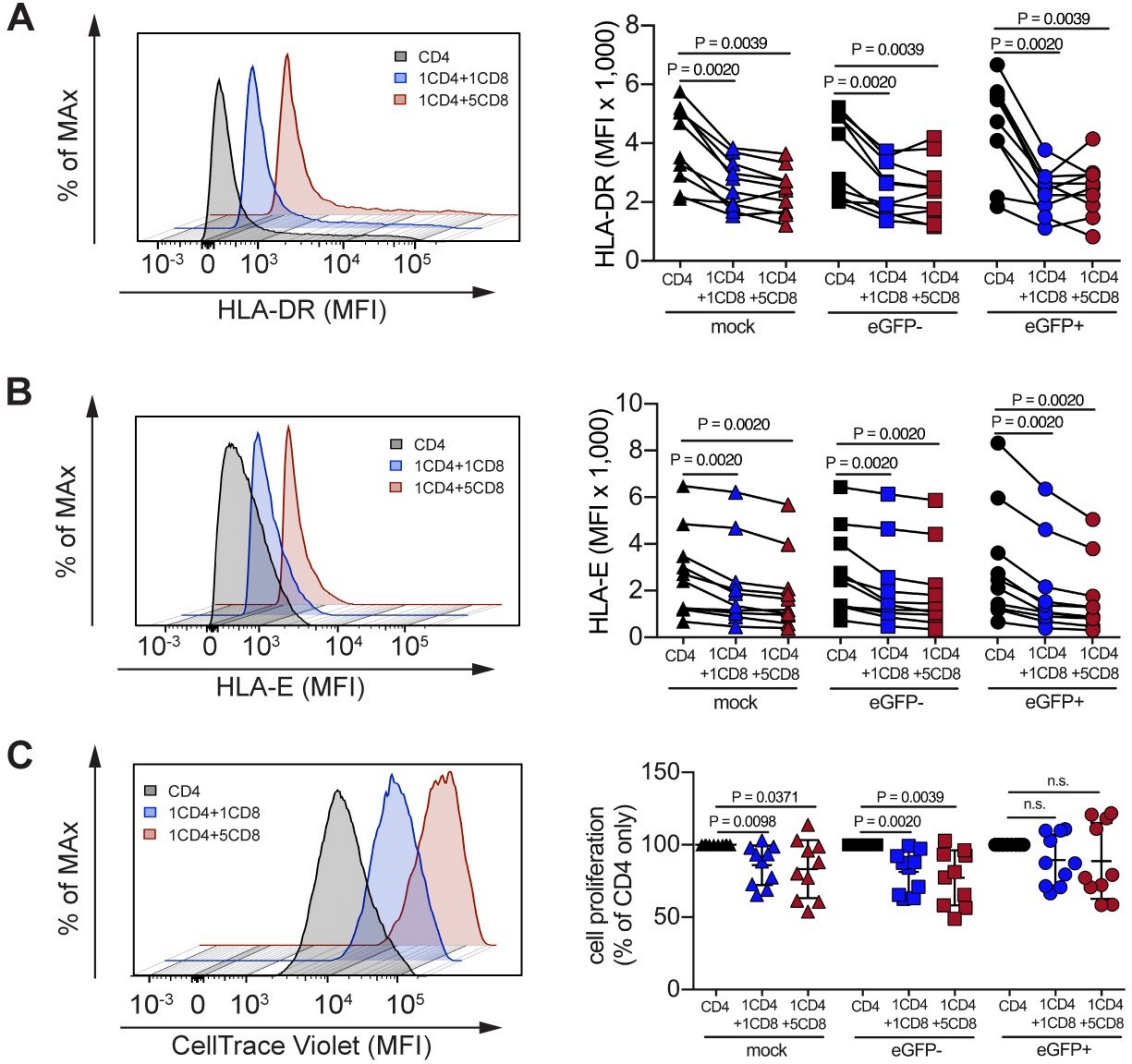
Effect of TCR-stimulated CD8^+^ T cells on HLA-DR, HLA-E and proliferation levels of uninfected and infected CD4^+^ T cells. Representative mean fluorescence intensity (MFI) of HLA-DR (**A**), HLA-E (**B**) and CellTrace Violet (**C**) is shown for non-productively infected and uninfected eGFP^-^CD4^+^ T cells from mono- and co-cultures in the multiple round infection assay. (**A** and **B**) The aggregate MFI data are also shown for HLA-DR and HLA-E of uninfected CD4^+^ T cells (mock; indicated by triangles), non-productively infected and uninfected CD4^+^ T cells (eGFP^-^; indicated by squares), and productively infected CD4^+^ T cells (eGFP^+^; indicated by circles) of 10 distinct subjects. Solid lines connecting the MFI values of activation markers (HLA-DR or HLA-E) from CD4 mono-culture wells (black), CD4/CD8 at 1:1 (blue) and 5:1 (red) E:T ratios from each subject represent the levels of suppression by CD8^+^ T cells. (**C**) Fold change in CellTrace violet MFI relative to CD4^+^ T cells alone followed by a f (x) = 1/x transformation are depicted for uninfected CD4^+^ T cells (mock; indicated by triangles), non-productively infected and uninfected CD4^+^ T cells (eGFP^-^; indicated by squares), and productively infected CD4^+^ T cells (eGFP^+^; indicated by circles) of 10 distinct subjects. Black lines represent the mean and standard deviation. Flow cytometric data for all the samples were performed on day 3 post- HIV infection, and comparisons between MFI of activation and proliferation markers on co-cultures with that of positive control wells (CD4^+^ T cells alone) were carried out using Wilcoxon matched-pairs signed rank test. See corresponding single cycle infections data of NL4-3_eGFP virus (S2B Fig) and NL4-3_D2eGFP virus (S2C Fig).

Along with HLA-DR, a classical cellular activation marker, we also measured HLA-E, based on previous observations by our group that all memory CD4^+^ T subsets (EM, TM, CM and SCM) exhibit substantial upregulation of surface HLA-E protein upon *in vitro* stimulation with CD3/CD28-beads to activate CD4^+^ T cells (S2A Fig). As shown in Fig 2A and 2B, CD8^+^ T cell co-culture was associated with a significant reduction of HLA-DR and HLA-E expression not only on productively infected (eGFP^+^) but also on non-productively infected and uninfected eGFP^-^CD4^+^T cells, with a pattern that was also observed in the matched uninfected control wells which had never been exposed to virus. Of note, similar results were observed with the single cycle infection assays (S2B and S2C Fig).

The finding that co-culture with CD8^+^ T cells reduces the level of CD4^+^ T cell activation suggests that mechanisms affecting lymphoproliferative responses might also be involved. Therefore, we next compared CD4^+^ T cell proliferation in infected CD4/CD8 co-culture to infected CD4^+^ T cells mono-culture. As shown in Fig 2C, we found that CD8^+^ T cells preferentially inhibited the proliferation of non-productively infected and uninfected eGFP^-^CD4^+^ T cells (mean fold change in CellTrace Violet MFI compared to CD4^+^ T cells alone, p = 0.0039). Even though the levels of proliferation on productively infected CD4^+^ T cells (eGFP^+^) were not consistently reduced (no significant changes) in the multiple-round infection, we found extended degrees of inhibition on productively infected CD4^+^ T cells (eGFP^+^) in the single cycle setting (p = 0.0027; S2B Fig and p = 0.0156; S2C Fig).

The matched uninfected control (mock infection) allowed us to address the question whether the virus itself was responsible for driving the CD8^+^ T cell-mediated suppressive activity or whether the observed effect was virus-independent. Consistent with the observations of lower activation levels, we found lower levels of proliferation among uninfected CD4^+^ T cells in the presence of CD8^+^ T cells as compared with those observed in uninfected CD4^+^ T cells of mono-cultures (Fig 2C). Altogether, these results strongly suggest that the observed CD8^+^ T cell-mediated suppression of HIV replication reflects, at least in part, a virus-independent immunoregulatory response that impacts overall CD4^+^ T cell activation and proliferation. This immunomodulatory effect of CD8^+^ T cells may not represent per se a directed antiviral response, and may contribute to CD4^+^ T cells transitioning more rapidly to a resting state that is less conducive to virus replication.

### CD8^+^ T cell-mediated virus suppression is non-cytolytic, MHC-independent, and associated with augmented secretion of IL-4, IL-5, IL-13 and sST2

**Fig 3:**
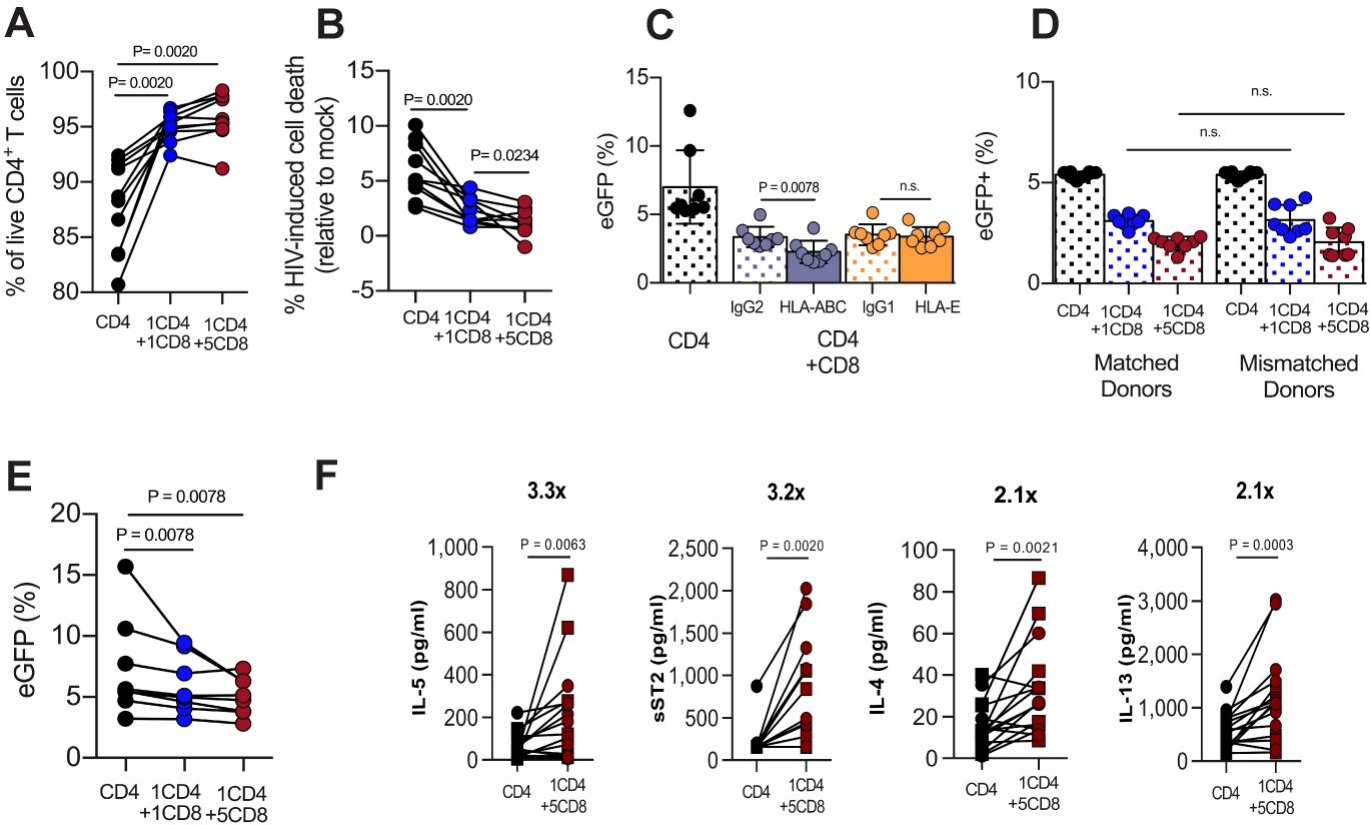
TCR-stimulated CD8^+^ T cells protect target cells from virus cytopathic effects, lack MHC-restriction, and is correlated with increased levels of Th2 cytokines. (**A-E**) I*n vitro* infections were conducted using the replication competent NL4-3_eGFP. (**A**) Frequencies of live CD4^+^ T cells were determined using a more stringent viability cutoff to include only cells that are negative for Live/Dead-Aqua dye labelling (live CD3^+^Vio^+^Red^-^ cells) in the infected mono—or co-culture wells (n = 10 subjects). (**B**) Frequencies of HIV-induced cell death were calculated by subtracting the frequency of live CD4^+^ T cells in the infected mono—or co-cultures wells by the frequency of live CD4^+^ T cells in the corresponding uninfected mono—or co-cultures wells (mock infection; n = 10 subjects). (**C**) Anti-pan HLA-ABC blocking mAb W6/32 (20 μg/ml), anti-HLA-E mAb 3D12 (20μg/ml), IgG2 (20 μg/ml) or IgG1 (20 μg/ml) were added to the CD4/CD8 co-cultures after infection. Bars represent mean frequencies of productively infected CD4^+^eGFP^+^ cells and black lines represent the standard deviation (n = 8 subjects). (**D**) Heterologous CD8^+^ T cells (mismatched donors) were added to CD4^+^ T cells and frequencies of productively infected CD4^+^eGFP^+^ cells were compared to the autologous co-cultures (matched donors). Bars represent mean values and black lines represent the standard deviation (n = 8 subjects). (**E**) In the transwell assay, infected CD4^+^ T cells were placed in the top chamber and autologous CD8^+^ T cells were placed in the bottom chamber. Frequencies of productively infected CD4^+^eGFP^+^ cells were compared between mono and- co-cultures (n = 8 subjects). **(F)** Supernatant levels of IL-5, sST2, IL-4, and IL-13 from CD4 mono-cultures and CD4/CD8 co-cultures at 5:1 (E:T). Experiments conducted with replication competent NL4-3_eGFP virus treated with protease inhibitor Darunavir are indicated by squares (n = 8 subjects), and experiments with replication-incompetent Env-defective NL4-3_D2eGFP are indicated by circles (n = 8 subjects). Flow cytometric data for all the samples were performed on day 3 post- HIV infection, and comparisons between co-cultures and mono-cultures were carried out using Wilcoxon matched-pairs signed rank test.

While MHC-I-restricted, HIV-1-specific cytotoxic CD8^+^ T cells are clearly present in PLWH [2–4, 6, 7, 43, 44] and can recognize and kill productively infected CD4^+^ T cells, the frequency of HIV-1-specific cytotoxic CD8^+^ T cells is thought to be extremely small in healthy, HIV-uninfected subjects[32, 33]. Based on this consideration, we reasoned that the possibility that CTL-mediated killing of infected CD4^+^ T cells is the mechanism underlying the observed antiviral activity of CD8^+^ T cells is highly unlikely. To test this prediction, we next measured the frequency of live CD4^+^ T cells and the frequency of virus-induced cell death relative to the uninfected cells in the control (mock infection), using the amine-reactive dye method that is based on the specific discrimination between cells that have lost membrane integrity and viable-live cells. As shown in Fig 3A and 3B, we found that co-culture with CD8^+^ T cells not only increased the frequency of live CD4^+^ T cells, but more importantly, mitigated virus-induced cell death in a dose dependent manner by 3- to 5-fold at 1:1 and 5:1 CD8:CD4 ratio, respectively. These results strongly support the hypothesis that the mechanism of virus suppression-mediated by TCR-activated CD8^+^ T cells from HIV-uninfected healthy donors is most likely non-cytolytic. In fact, our results indicate that the presence of CD8^+^ T cells promoted CD4^+^ T cell survival, possibly by protecting the pool of productively infected CD4^+^ T cells from virus cytopathic effects.

Since the preceding results are in agreement with the hypothesis that HIV-1 suppression is mediated by an innate virus-independent, immunoregulatory mechanism, we next investigated whether this suppressive activity was dependent of the MHC-based T cell response. To this end, we treated CD4/CD8 T cell co-cultures with the anti-pan HLA-ABC blocking mAb W6/32 (20 μg/ml), or the anti-HLA-E mAb 3D12 (20μg/ml), and compared those results to co-cultures from the same donors treated with the correspondent isotype control antibodies. Furthermore, we also assessed the suppressive activity in a heterologous co-culture system in which CD4^+^ and CD8^+^ T cells from different donors were mismatched. Interestingly, we found that HLA-ABC and HLA-E blockage (Fig 3C), as well as co-culture with heterologous CD8^+^ T cells (Fig 3D), failed to remove the HIV suppression, thus further supporting the hypothesis of a MHC-independent, CD8^+^ T cell-mediated mechanism of suppression.

To next investigate whether the viral suppressive effect of CD8^+^ T cells requires cell-to-cell contact, we used a transwell (0.4 μm) cell culture system to physically separate effector and target cell populations. We found that soluble factors had a moderate effect on HIV expression with a mean suppression of 19% and 31% at 1:1 and 5:1 E:T (Fig 3E), whereas contact between CD4 and CD8^+^ T cells resulted in 56% and 73% at 1:1 and 5:1 E:T reduction in HIV expression (Fig 1B). These results indicate that soluble factors may be involved in the CD8^+^ T cell-mediated suppression of HIV production, even though cell-to-cell contact and/or soluble factors with a short half-life that require close proximity between CD4 and CD8^+^ T cells likely mediate the most potent suppressive activity on HIV production. In fact, previous reports have demonstrated that non-cytolytic mechanisms of HIV suppression by CD8^+^ T cells include the block of virus spread and entry via production of chemokines such as CCL3/MIP-1a, CCL4/MIP-1b, and CCL5/RANTES[13]; however, to date, no CD8^+^ T cell-secreted factor has been identified as the key mediator of the HIV-1 transcription-suppressing activity of CD8^+^ T cells. To identify potential soluble factors associated with HIV suppressive activity in our experimental system of single round infection, we first used a global approach of membrane-based cytokine array to screen the secretome profile of CD4/CD8 co-cultures versus CD4 mono-cultures. We found that 18 of the 274 tested soluble factors were present at higher levels in the 5:1 (E:T CD8/CD4) supernatants than in the CD4 mono-culture supernatants (p<0.05) (S1 Table). Interestingly, the preferential enrichment of the T helper 2 (Th2) cytokines IL-4, IL-5, IL-13, and sST2 was further confirmed using cytometric bead array (CBA, Fig 3F), suggesting that these cytokines may play a role in mediating the observed suppression of HIV production in the CD4/CD8 co-cultures.

### CD8^+^ T cell-mediated virus suppression increases the frequency of infected CD4^+^ T cells with inducible virus

**Fig 4:**
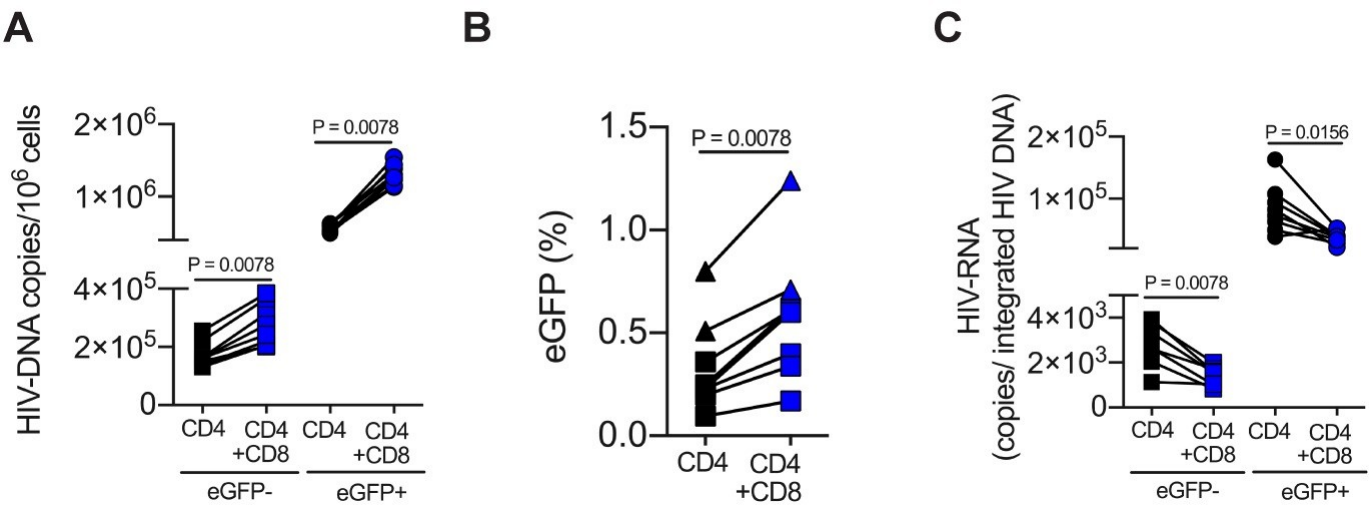
Infected CD4^+^ T cells co-cultured with CD8^+^ T cells accumulate reservoir-harboring cells compared to CD4^+^ T cells alone. All *in vitro* infections were conducted using the replication competent NL4-3_eGFP under single cycle condition with protease inhibitor (Darunavir), with the exception of 2 donors who were infected with the (Env)-defective NL4-3_eGFP in the inducible provirus experiment (indicated by triangle symbol). CD4^+^ T cells from mono- and co-cultures with CD8^+^ T cells at 1:1 E:T ratio were FACS-sorted based on eGFP^+^ and eGFP^-^ expression 3 days post-infection (see **S3 Fig**). Data from 8 subjects are shown. (**A**) Integrated HIV-DNA copies/10^6^ cells are shown for sorted non-productively infected and uninfected (eGFP^-^) and sorted-productively infected (eGFP^+^) CD4^+^ T cells from mono—or co-cultures. (**B**) Sorted eGFP^-^CD4^+^ T from mono—and co-cultures were reactivated by CD3/28 stimulation three days post-infection and controlled for i) de novo infection with protease inhibitor (Darunavir) and ii) pre-integration events with integrase inhibitor (Dolutegravir). Inducible provirus was determined by the frequency of eGFP^+^ cells 24 hours post-reactivation using flow cytometry. (**C**) Levels of HIV transcripts on a per-integrated-provirus basis were calculated by dividing the copy numbers of cell-associated multiply-spliced Tat-Rev HIV-RNA per copy numbers of integrated HIV-DNA per cell. Comparisons between co-cultures and mono-cultures were carried out using Wilcoxon matched-pairs signed rank test.

Since we demonstrated that CD8^+^ T cells preferentially reduce the activation and proliferation levels of CD4^+^ T cells, we next tested how the presence of CD8^+^ T cells specifically affects the fraction of CD4^+^ T cells infected with HIV. Using a single cycle infection assay, we blocked the replication-competent NL4-3_eGFP virus with protease inhibitor Darunavir, and sorted the non-productively-infected and uninfected (eGFP^-^) and productively-infected (eGFP^+^) CD4^+^ T-cell populations from CD4/CD8 co-cultures or CD4 mono-cultures three days post infection to assess: (i) the amount of integrated HIV-DNA, (ii) the frequencies of inducible provirus by reactivation of sorted eGFP^-^CD4^+^ T population, and (iii) the number of HIV transcripts on a per-integrated-provirus basis. Importantly, we detected an average 1.6- to 2.4-fold increase of integrated HIV-DNA copy numbers in CD4^+^ T cells when CD8^+^ T cells were present (p = 0.0078 for eGFP^-^, and p = 0.0078 for eGFP^+^; Fig 4A). We also found that the pool of CD4^+^ T cells that harbor inducible provirus in the eGFP^-^ CD4^+^ T cell population from the co-culture with CD8^+^ T cells was also significantly increased by 75% (p = 0.0078; Fig 4B), thus confirming that CD8^+^ T cells increase the frequency of infected CD4^+^ T cells with inducible virus. In addition, we quantified virus transcriptional silencing on a per-integrated-provirus basis, with an index expressed as copy numbers of multiply-spliced Tat-Rev HIV-RNA per copy of integrated HIV-DNA per cell in the same donor. Consistent with the previous results using the NL4-3_D2eGFP virus which demonstrated suppression of HIV- LTR driven expression (Fig 1D), we found that CD8^+^ T cells significantly reduced the levels of HIV-RNA transcripts on a per-integrated-provirus basis (56%, p = 0.0078 for eGFP^-^, and 54%, p = 0.0156 for eGFP^+^; Fig 4C). Taken together, these results support the hypothesis that CD8^+^ T cell-mediated suppression selectively favors the survival of infected CD4^+^ T cells with inducible virus. This effect results in a significant enrichment of the frequency of CD4^+^ T cells that harbor a larger proviral load and inducible virus but produce little to no virions due to a CD8^+^ T cell-mediated transcriptional silencing.

### CD8^+^ T cell-mediated virus suppression changes the transcriptome profile of infected CD4^+^ T cells

**Fig 5:**
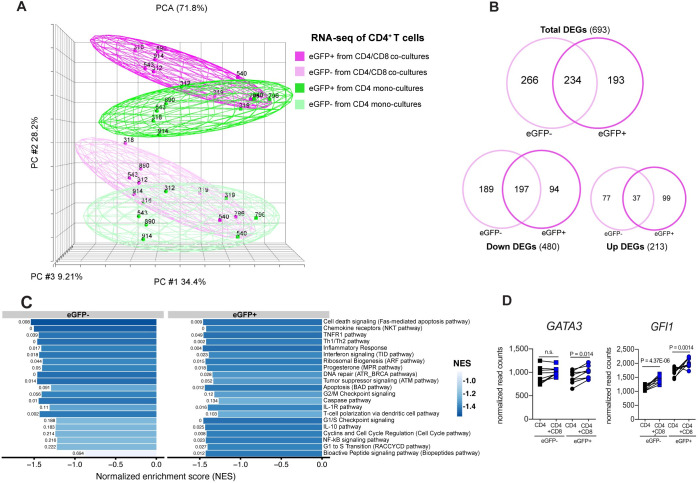
Transcriptome analysis of infected CD4^+^ T cells reveals systematic immune-suppressive activity by CD8^+^ T cells. All *in vitro* infections were conducted using the replication competent NL4-3_eGFP under single cycle condition with protease inhibitor (Darunavir). CD4^+^ T cells from mono- and co-cultures with CD8^+^ T cells at 1:1 E:T ratio were FACS-sorted based on eGFP^+^ and eGFP^-^ expression 3 days post-infection (see **S3 Fig**). Data from 8 subjects are shown. (**A**) Principal-component analysis (PCA) of RNA-seq data shows CD4^+^ T sorted subsets derived from CD4 mono-cultures (eGFP^-^, light green; eGFP^+^; dark green) and CD4^+^ T sorted subsets derived from CD4/CD8 co-cultures (eGFP^-^, light magenta; eGFP^+^; dark magenta). Each point represents a sample labelled with the donor ID. Female subjects are indicated by circles, and male subjects are indicated by squares. (**B**) Venn diagrams demonstrating differentially expressed genes (DEGs) in eGFP^-^ and eGFP^+^CD4^+^ T subsets in response to CD8^+^ T cells (DESeq2: padj <0.05 and fold change threshold >1.5 (0.585 log) or <0.67 (-0.585 log). Intersection shows DEGs in common between eGFP^-^ and eGFP^+^CD4^+^ T subsets in response to CD8^+^ T cells. (**C**) Barplot shows the pathways significantly downregulated in eGFP^-^ and eGFP^+^CD4^+^ T subsets by the CD8^+^ T cells obtained from the MSigDB database BioCarta collection, GSEA. A normalized enriched score (NES) lower than 0 corresponds to a pathway for which member genes are downregulated in CD4/CD8 co-cultures. The 21 pathways depicted presented a NES < -1.4 in either eGFP^-^ or eGFP^+^CD4^+^ T subsets. The color scale denotes the maximum and minimum normalized enrichment score (NES), and the nominal p value alongside the bars, estimates the statistical significance of the enrichment score for a single gene set. For leading edge genes of Fas, Th1/Th2, Inflammatory and G2/M pathways (see **S4 Fig**). (**D**) Effect of CD8^+^ T cells on *GATA3* and *GFI1* transcriptional regulator genes associated with the Th2 pathway of CD4^+^ T helper differentiation (DESeq2 library size normalized read counts).

To identify previously unrecognized cellular pathways that are associated with virus suppression by CD8^+^ T cells, we next assessed the transcriptional profile of eGFP^-^ and eGFP^+^CD4^+^ T sorted subsets from CD4 mono—and CD4/CD8 co-cultures at three days post-infection. As shown in Fig 5A, we first conducted a covariance principal component analysis (PCA) using the 500 genes that display the most variance across all samples, and observed that: (i) inter-donor variability accounts for the largest variance (35%), with 5 out of 5 female donors cluster separate from 3 out of 3 male donors along the first principal component axis (PC #1); (ii) the viral production (i.e., expression of eGFP) represented the second largest source of variance (28.2%), with productively infected eGFP^+^CD4^+^T cell populations clearly distinguished from non-productively infected and uninfected eGFP^-^CD4^+^T cell populations, and independent of donor variability along the second principal component axis (PC #2); and (iii) the presence of CD8^+^ T cells results in a consistent shift per sample in the first three principal components to the left in PC #1, up in PC #2 and back in PC #3, with transcriptomic profiles of the eGFP^-^ and eGFP^+^CD4^+^ T populations from CD4/CD8 co-cultures clustered away from the eGFP^-^ and eGFP^+^CD4^+^ T populations from the CD4 mono-cultures. Overall, these results indicate a clear and comparable effect of CD8^+^ T cells on the transcriptional programming of CD4^+^ T undergoing either productive or non- productive HIV-1 infection.

To better characterize the CD8^+^ T cell mediated- effect on sorted eGFP^-^ and eGFP^+^CD4^+^ T subsets, we next performed RNA-seq differential expression and statistical analysis between mono and-co-cultures using the DESeq2 package[45](fold change threshold >1.5 or <0.67; padj <0.05). Consistent with the results captured by the first three principal components, we confirmed that CD8^+^ T cells regulated the transcriptional activity of the productive and non-productive infection in a comparable way, as both eGFP^-^ and eGFP^+^CD4^+^ T populations shared approximately 50% of the differentially expressed genes (DEGs) (Fig 5B). Furthermore, we found no evidence of genes whose expression is modulated in opposite directions (i.e., upregulated versus downregulated) between productive and non-productive infection in the presence of CD8^+^ T cells (S8 Fig). Interestingly, we found that co-culture with CD8^+^ T cells resulted in 480 downregulated and only 213 upregulated gene transcripts, thus indicating a net suppression of the overall transcriptional activity of CD4^+^ T cells (Fig 5B). Overall, these results indicate that co-culture with activated CD8^+^ T cells has a profound effect on the transcriptional profile of both productively and non-productively HIV-1-infected CD4^+^ T cells that appears to result in a global reduction of the transcriptional activity of these cells.

To further characterize the distinct biological pathways regulated in response to CD8^+^ T cells, we next performed gene set enrichment analysis (GSEA) with 217 curated canonical pathways derived from the BioCarta database[46]. As shown in Fig 5C, we observed a significant reduction of several signaling pathways related to cell death, including CD95/Fas, Tumor necrosis factor receptor 1 (TNFR1), Bcl-2-associated death promoter (BAD) and Caspase, therefore consistent with the observation that CD8^+^ T cells suppressed HIV expression via molecular pathways that do not involve death of CD4^+^ T cells. In addition, we observed a downmodulation of gene expression pathways associated with: (i) CD4^+^ T cell differentiation such as Th1/Th2 response, chemokine receptors (NKT pathway), cell polarization (DC pathway), cytokine and inflammatory response such as IL-10, ILR1, IFN (TID) and NF-kb pathways, thus consistent with cells transitioning from a polarized phenotype to relative quiescence; and (ii) CD4^+^ T cell proliferation and DNA repair such as ARF, ATR and BRCA, G1, G2, and Ras pathways thus confirming the inhibitory effect of CD8^+^ T cells on CD4^+^ T cells proliferation (Fig 5C). Consistent with the Th2 bias suggested by the secretome profile of co-culture experiments, we found that the co-culture with activated CD8^+^ T cells resulted in a significant upregulation of transcriptional regulator genes associated with the Th2 pathway of CD4^+^ T helper differentiation (*GATA3*, padj = 0.014 for eGFP^+^; and *GFI1*, padj = 4.37x10^-6^ for eGFP^-^ and padj = 0.0014 for eGFP^+^; Fig 5D). Altogether, these data support our hypothesis that CD8^+^ T cells are capable of suppressing HIV replication through non-cytolytic mechanisms that involved a net downmodulation of CD4^+^ T cell activation as well as a significant upregulation of genes associated with Th2 differentiation.

## Discussion

The mechanisms underlying the CD8^+^ T cell-mediated control of HIV replication likely involve non-mutually exclusive cytolytic and non-cytolytic activities, whose relative *in vivo* contribution to the antiviral effect of CD8^+^ T cells remains incompletely understood. A better characterization of these mechanisms may reveal novel targets for immune-based interventions that can reduce or prevent HIV replication in PLWH. The recent recognition that CD8^+^ T lymphocytes cooperate with ART to maintain virus suppression by inhibiting virus production from latently-infected CD4^+^ T cells emphasizes the importance of understanding the antiviral roles of these cells in the setting of HIV cure research[20, 21].

In this present study, we investigated the role of non-cytolytic CD8^+^ T cell-mediated mechanisms of HIV suppression by using an *in vitro* experimental system consisting of infected CD4^+^ T cells co-cultured with autologous activated CD8^+^ T cells derived from healthy HIV-uninfected subjects in which only an extremely small fraction of naïve HIV-specific T cell precursors is present. In addition, we conducted experiments using CD4^+^ and CD8^+^ T cells from different donors in which the lack of classical MHC-I restriction (Fig 3D) would not enable a classical HIV-specific CTL activity[47–49]. Since CD8^+^ T cells may block HIV/SIV spread from cell- to-cell *via* CCR5-binding chemokines (i.e., MIP-1a/CCL3, MIP-1b/CCL4, and RANTES/CCL5)[1, 13], we also used viruses which were limited to a single cycle of infection. This latter experimental system allowed us to distinguish between antiviral effects that inhibit viral spread by blocking viral entry and those that affect post-entry processes during a single cycle of virus replication (Fig 1B and 1D). To the best of our knowledge, the current study represents the most comprehensive investigation focusing on the potential antiviral role of CD8^+^ T cells occurring via transcriptional silencing of HIV.

Our experimental investigation revealed that TCR-activated CD8^+^ T cells, but not B cells or monocytes (Fig 1C), exert a potent block of virus replication that is consistent with transcriptional inhibition of HIV expression in infected CD4^+^ T cells. In this context, we assessed changes in HIV-LTR driven gene expression using an Envelope-defective NL4-3 reporter virus that encodes a particularly sensitive short-lived D2eGFP variant, and also measured cell-associated Tat/rev multiply spliced HIV-RNA. The generated results demonstrate that CD8^+^ T cell-mediated activity (i) suppresses the LTR-dependent viral gene expression as quantified by D2eGFP levels(Fig 1D), and (ii) interferes with the production of multiply spliced HIV-1 transcripts(Fig 4C). Since similar degrees of virus transcriptional silencing were observed in both productive and non-productive infection, we propose that non-cytolytic CD8^+^ T cells might act as virus-independent innate effectors that regulate CD4^+^ T- cell-intrinsic signaling pathways rather than specifically suppress the viral transcription itself. In line with this hypothesis, we further confirmed that CD8^+^ T cells significantly reduced the CD4^+^ T-cellular levels of activation and proliferation in both productive and non-productive infection, an effect which occurred independently of the presence of HIV infection (i.e., in both infected cell cultures as well as uninfected matched controls) (Fig 2). Additionally, a similar magnitude of inhibition in regard to cellular activation and proliferation was found when cells from single or multiple-round of infections were analyzed(S2B and S2C Fig). Therefore, these results identify an important regulatory effect of non-cytolytic CD8^+^ T cells on activated CD4^+^ T cells that drives CD4^+^ T cells into a more resting state that is less conducive to HIV replication. It is important to note that the observed immune-regulatory effect of CD8^+^ T cells on CD4^+^ T cell activation is not per se surprising as a similar effect has been shown in humans and murine models of autoimmunity, transplant rejection, and anti-tumor immune responses[36–41, 50, 51].

Since experiments conducted by physically separating CD4^+^ and CD8^+^ T cells suggested that the CD8^+^ T cell-mediated transcriptional signaling is caused, at least in part, by soluble factors(Fig 3E), we conducted a comprehensive analysis of the secretome in our co-cultures. This analysis was consistent with the establishment of a potentially immune-modulatory environment characterized by the presence of Th2 cytokines and anti-inflammatory mediators (S1 Table). This intriguing conclusion was confirmed by a comprehensive analysis of the transcriptome of co-cultured CD4^+^ T cells that revealed a profound downmodulation of multiple gene sets of inflammation (such as *IL1A*, *TNF*, and *IFNB1*); cell death (e.g., *CASP-3*, *-6*, *-8*, *-10*, *FAS*, and *FASL*); proliferation and DNA repair (*CDK1* and *CDC25A*) in concert with the upregulation of transcriptional regulator genes of Th2 differentiation (*GATA3* and *GFI1*); but counter-regulation of Th1 responses (*IFNG*, *IL12A*, *IL12RA*, and *IL12RB2*) (Fig 5D and **S4 Fig**). In line with our transcriptome and secretome findings, a recent study by Cyktor et al., found that IL-4, and IL-13 were associated with the levels of intact proviral DNA in PLWH with a median of 7 years on ART[52].

Interestingly, neither the addition of IL-4, -5, -13, -33R cytokines to infected CD4^+^ T cells nor the blocking of those Th2 cytokines in CD4/CD8 co-cultures fully recapitulated the features of CD8^+^ T cell mediated suppressive activity of viral transcription (S6 and S7 Figs). As such, these results further support the hypothesis that CD8-to-CD4 contact mediates the most potent suppressive activity on HIV production, and suggest that the observed higher levels of Th2 cytokines reflect a cell differentiation signature rather than a direct role for these molecules in establishing the suppressive phenotype.

Of note, the global transcriptome profile of infected CD4^+^ T cells clearly distinguished the CD8^+^ T cell-mediated suppressive activity between sexes, showing that substantial changes are stronger in the female donors (PCA, Fig 5A). This finding is consistent with the existing literature reporting that sex differences in immune responses result in differential susceptibility of males and females to autoimmune diseases, malignancies and infectious diseases, as well as affecting the outcome of vaccination (reviewed in [53]). In the context of HIV infection, several studies have reported that women exhibit lower HIV RNA levels and improved ART-mediated viral suppression[54] suggesting different immune modulation between sexes, which deserve to be further investigated.

Collectively, our unbiased global analysis of the secretome and transcriptome should not be taken as a conclusion, but rather as a hypotheses-generating study that should prompt additional in-depth investigation of this intriguing and previously unrecognized role of non-cytolytic CD8^+^ T cells in contributing to the control of HIV infections. With this caveat in mind, the hypothesis that emerges from our comprehensive set of data is that activated CD8^+^ T cells can modulate the level of CD4^+^ T cell activation and proliferation in a manner that involves the induction of some levels of Th2 differentiation and can be considered both innate (i.e., non-MHC-dependent) and virus-independent (i.e., not related to the presence of HIV) in its nature. In this scenario, the observed non-cytolytic activity, immune-modulatory activity of CD8^+^ T cells has a significant impact on HIV replication by reducing and silencing its transcription within infected CD4^+^ T cells.

In the setting of untreated HIV-1 infection (i.e., absence of ART), all antiviral effects of CD8^+^ T cells, including (i) CTL activity, (ii) release of β-chemokines that block virus entry, and (iii) the above-described virus transcriptional silencing, can contribute to the control of virus replication and therefore may be involved in generating the so-called “elite controller” phenotype in which rare PLWH show extremely low viremia in absence of ART[8]. However, in the setting of ART-treated HIV infection, both CTL activity and block of virus entry are likely to result in a net decline of the total number of infected cells, while transcriptional silencing of HIV could result in an increased number of latently-infected cells, and therefore paradoxically favor the persistence of the virus reservoir. The likelihood of this outcome is emphasized by the observation that co-culture with activated CD8^+^ T cells promotes the survival of infected CD4^+^ T cells (Fig 3A and 3B). At this time, it remains unclear whether the presence of CD8^+^ T cells promotes the survival of infected CD4^+^ T cells simply by reducing the level of virus production (and therefore the risk of direct cytopathicity) or by increasing the resistance of CD4^+^ T cells to the cytopathic effects of HIV. Importantly, the HIV transcriptional silencing observed in infected CD4^+^ T cells co-cultured with activated CD8^+^ T cells is not associated with a decline of the copy numbers of integrated HIV-DNA (Fig 4A) or the frequencies of inducible provirus (Fig 4B). While our experimental system did not directly address the impact of activated CD8^+^ T cells in the establishment and maintenance of HIV latency in CD4^+^ T cells, it did not escape our attention that this non-cytolytic activity of CD8^+^ T cells may increase the *in vivo* pool of latently infected CD4^+^ T cells under ART and therefore represent a key, previously unrecognized obstacle to the elimination of the virus reservoir and the eradication of HIV infection. If this hypothesis is confirmed by further studies, blocking the CD8^+^ T cell-mediated transcriptional silencing of HIV may be explored in future immune-based interventions to cure the infection.

In summary, this study provides strong evidence that an innate, non-MHC-dependent, non-cytolytic antiviral activity of CD8^+^ T cells potently suppress HIV replication through silencing of the LTR-dependent viral transcription. This antiviral effect is related to virus-independent immune-modulatory mechanisms that decrease CD4^+^ T cell activation and proliferation, increase CD4^+^ T cell survival and influence the differentiation of CD4^+^ T cells towards a Th2 profile. In the setting of ART-treated HIV-infection, the CD8^+^ T cell mediated transcriptional silencing of HIV may selectively favor the survival of infected CD4^+^ T cells harboring integrated, inducible virus, and therefore represent a previously unrecognized obstacle to HIV eradication.

## Materials and methods

### Human subjects

Peripheral blood was collected from individuals classified as HIV-negative (n = 42 healthy adult subjects; 20 males and 22 females) recruited from the LifeSouth Community Blood Centers (Atlanta, GA).

### Ethics statement

All study subjects enrolled in this research provided written informed consent as per protocols approved by the Emory University Institutional Review Board (IRB) IRB00045821.

### Enrichment and stimulation of primary cells

Peripheral blood mononuclear cells (PBMCs) were obtained using Ficoll-Hypaque density gradient centrifugation. CD4^+^ and CD8^+^ T cells were purified by negative selection (Miltenyi Biotec) and stimulated with human CD3/CD28/CD2 antibody-coated beads (Miltenyi Biotec) at 1:1 bead-to-cell ratio, in the presence of 50 IU/ml of rhIL-2 (Miltenyi Biotec) for three days. CD19^+^ B cells were purified by positive selection (Miltenyi Biotec) and stimulated with 8 IU/ml of CD40L (Miltenyi Biotec) in the presence of 50 IU/ml of IL-4 (Miltenyi Biotec) for three days. CD14^+^ monocytes were purified by positive selection (Miltenyi Biotec) and stimulated with 100 ng/ml of LPS (eBioscience) and 500 ng/ml of R848 (InvivoGen) for three days. Primary cells were maintained in RPMI 1640 medium (Corning) supplemented with 10% FBS, 100 U/ml of penicillin, 100 μg/ml of streptomycin, and 2 mM of L-glutamine at 37°C/5% CO2.

### Virus production

A replication-competent and an Envelope (Env)-defective NL4-3 reporter virus expressing eGFP under control of the HIV- 1 LTR were kindly provided by F Kirchhoff[55] (Ulm University Medical Center, Ulm, Germany). Envelope (Env)-defective NL4-3_eGFP was then complemented in trans with a dual-tropic Env (pSVIII-92HT593.1) obtained through the NIH-NIH AIDS Reagent Program from Dr. Beatrice Hahn (cat# 3077). A variant of the (Env)-defective NL4-3 IRES-eGFP reporter virus encoding the short-lived D2eGFP version of the protein was generated as previously described[35] by inserting the protein-destabilizing mouse ornithine decarboxylase (MODC) domain at the Emory Custom Cloning Core Division using standard cloning techniques. Viral stocks were produced by single or co-transfection of Lenti-X 293T cells (STR authenticated; Takara Bio) with the respective HIV-1 constructs, using FuGene6 (Promega) in OptiMEM (Thermo Fisher). Lenti-X 293T cells were maintained in Dulbecco’s modified Eagle medium (DMEM) (Gibco) supplemented with 10% FBS, 100 U/ml of penicillin, 100 μg/ml of streptomycin, 2 mM of L-glutamine at 37°C/5% CO2.

### Dual-color co-culture assays

Target CD4^+^ T cells were labelled with CellTrace Violet dye (Vio) and effector CD8^+^ T, CD19^+^ B, or CD14^+^ monocytes were separately labelled with CellTrace Red dye (Red), prior to infection. Briefly, TCR-activated CD4^+^ T cells were labelled with 5 μM of CellTrace Violet dye (Vio) (Thermo Fisher) per 10x10^6^ cells and were subsequently infected by spinoculation (2h, 37°C, 2,500 rpm) using 100 μl of virus stock/0.6x10^6^ cells in flat-bottom 96-well plates. For uninfected negative controls (mock infection) we added 100 μl of cell culture media/0.6x10^6^ cells. In parallel, autologous TCR-activated CD8^+^ T cells, activated B cells or activated monocytes were labelled with 5 μM of CellTrace Red dye (Red) (Thermo Fisher) per 10x10^6^ cells and added to the Vio^+^CD4^+^ T cells at 1:1 and 5:1 effector-to-target cell (E:T) ratios in the presence of 50 IU/ml of rhIL-2 after infection in 48-well plates. Heterologous co-culture assays were set up as described above, except that CD8^+^ T cells were from mismatched donors. In transwell assays, Vio^+^CD4^+^ T cells were placed in the top chamber and Red^+^CD8^+^ T cells in the bottom chamber of 6.5 mm plates with 0.4 μm pore membrane insert (Corning). In all experiments, the total number of cells were kept constant at a cell density of 0.6x10^6^ cells/ml/well in mono-cultures and co-cultures to control for variations in media consumption between conditions. To block HLA-ABC and HLA-E signaling, antibodies were 30KDa-filtered in phosphate-buffered solution, pH 7.2 (Amicon, Millipore Sigma) and subsequently 20 μg/ml of anti-pan HLA-ABC blocking mAb W6/32 (BioLegend) or anti-HLA-E mAb 3D12 (BioLegend) were added after infection for the duration of the co-cultures, respectively. Corresponding isotype control antibodies (BioLegend) were similarly purified and used as negative controls for comparison. Where mentioned, single cycle infections were performed either by blocking the production of mature particles from replication-competent NL4-3_eGFP virus with 100 nM of protease inhibitor (Darunavir; Janssen Sciences), or by simply using a replication-incompetent, Envelope (Env)-defective NL4-3_eGFP complemented in trans with a dual-tropic Env.

### Flow cytometry analyses

For assessment of cell activation, proliferation, survival and eGFP expression flow cytometric analyses were performed in mono- or co-cultures three days post-infection. Cells were first stained with cell viability dye (Live/ Dead Fixable Aqua from Thermo Fisher) for 10 min and then stained with monoclonal antibodies to CD4-BV650 (clone OKT4), CD8-PE-Cy5 (clone RPA-T8) and HLA-E-PE (clone 3D12) from Biolegend; CD3-PE-CF594 (clone SP34-2) and HLA-DR-PE-Cy7 (clone G46-6) from BD Biosciences; and HLA-ABC- PerCP-eFluor 710 (clone W6/32) from Thermo Fisher for 30 min. At least 100,000 live CD3^+^ T cells were acquired on a LSR II flow cytometer (BD Biosciences), equipped with FACS Diva software. Analysis of the acquired data was performed using FlowJo software (version 10.1r5).

### Cell sorting

For assessment of cell host and viral RNA, as well as integrated and inducible provirus, FACS-sorting of productively infected (eGFP^+^) and non-productively infected as well as uninfected CD4^+^ T cells (eGFP^-^) was performed three days post-infection using the replication competent NL4-3_eGFP under single cycle condition with 100 nM Darunavir. Mono- and co-cultures were stained as previously described with Live/Dead-Aqua, and with CD3, CD4 and CD8 antibodies. Cells were initially gated on singlets (FSC-H versus FSC-A), on the basis of light scatter (SSC-A versus FSC-A), and followed by positive staining for CD3 and negative staining for Live/Dead Aqua. Effector CD8^+^ T cells were gate out based on their positive staining for Cell Trace Red. The target populations were then gated on their positive staining for Cell Trace Violet followed by a negative staining for CD8 on a CD8 versus CD4 plot to include the Nef-down-modulated CD4 negative population. The uninfected (mock) wells were used as negative controls to draw a gate for the HIV-infected (eGFP) wells, which were subsequently sorted as productively infected eGFP^+^CD4^+^ T cell population derived from live CD3^+^Vio^+^Red^-^CD8^-^eGFP^+^ and non-productively infected as well as uninfected eGFP^-^CD4^+^ T cell population derived from live CD3^+^Vio^+^Red^-^CD8^-^GFP^-^ (**S3 Fig**). Cell sorting was performed by the Emory Vaccine Center’s Flow Cytometry Core, cells were acquired on a BSL-3 FACS Aria II cell sorter (BD Biosciences) equipped with FACS Diva software.

### Quantification of inducible provirus

1 x 10^6^ eGFP^-^CD4^+^ T cells from the mono—and co-cultures were FACS-sorted as shown in **S3 Fig**, and were re-stimulated with human CD3/CD28/CD2 antibody-coated beads (bead-to-cell ratio 1:1) or, with plate-bound anti-CD3 (clone OKT3; eBioscience) and soluble anti-CD28 (clone 28.2; eBioscience) at 2 μg/ml, in the presence of 50 IU/ml of rhIL-2 (Miltenyi Biotec), 100 nM Darunavir (Janssen Sciences) and 1 μM Dolutegravir (Viiv Healthcare), on day 3 post-HIV infection. Inducible provirus was determined 24 hours post-reactivation by the frequency of eGFP^+^ cells using flow cytometry. Analysis of the acquired data was performed using FlowJo software (version 10.1r5).

### Purification of total viral nucleic acids

0.5 x 10^6^ eGFP^-^CD4^+^ T cells and at least 0.5 x 10^5^ eGFP^+^CD4^+^ T cells were FACS-sorted from the mono- and co-cultures as shown in **S3 Fig**, pelleted, disrupted by Qiagen RLT buffer with 1% of 2-Mercaptoethanol (BME), and stored at -80°C immediately. Total viral nucleic acids were extracted using the QIAamp Viral RNA Mini Kit (Qiagen) per the manufacturer’s instructions.

### Quantification of integrated HIV-DNA

A real time nested polymerase chain reaction (PCR) was performed to determine the frequency of cells harboring integrated HIV DNA (adapted from[56]). Briefly, 2.5 μl of the purified viral acid nucleic was used in a 25 μl first-round PCR reaction. The second-round of PCR reaction was performed in a final volume of 20 μl containing 6.4 μl of a 1/10 dilution of the first PCR products. Integrated HIV-DNA was amplified using primers and probe specific for the LTR-Gag region and for the human Alu sequences. In all PCR reactions, primers and probe specific for the CD3 gene were added to precisely quantify the cell input. The number of copies of integrated HIV-1 DNA was calculated by using serial dilutions lysed ACH-2 cells as a standard curve. Results were expressed as numbers of HIV copies per million cells.

### Quantification of cell-associated Tat/rev multiply spliced HIV RNA

A real time quantitative reverse transcription polymerase chain reaction (qRT-PCR) was performed to determine the frequency of cells harboring Tat/rev multiply spliced HIV RNA (adapted from[57]). Briefly, 1μl of the purified viral acid nucleic was used in a 10 μl first-round RT-PCR. The second-round of PCR reaction was performed in a final volume of 10 μl containing 1μl of a 1/10 dilution of the first PCR products. Multiply spliced HIV RNA was amplified using primers and probe specific for the tat/rev region. RNA copy numbers were calculated based on the RNA controls from an *in vitro* transcription of target DNA (T7-Tat/Rev).

### RNA-seq library preparation

RNA-seq analysis was conducted at the Yerkes Nonhuman Primate Genomics Core Laboratory(http://www.yerkes.emory.edu.proxy.library.emory.edu/nhp_genomics_core/).

50,000 eGFP^-^ and eGFP^+^CD4^+^ T cells were FACS-sorted from mono- and co-cultures as shown in **S3 Fig**. Sorted populations were stored in 350 μl of RLT buffer with 1% of 2-Mercaptoethanol (BME) at -80°C, purified using Qiagen RNeasy Micro columns, and RNA quality was assessed using an Agilent Bioanalyzer. Ten nanograms of total RNA was used as input for cDNA synthesis using the Clontech SMART-Seq v4 Ultra Low Input RNA kit according to the manufacturer’s instructions. Amplified cDNA was fragmented and appended with dual-indexed bar codes using the Illumina NexteraXT DNA Library Preparation kit. Libraries were validated by capillary electrophoresis on an Agilent 4200 TapeStation, pooled, and sequenced on an Illumina HiSeq3000 at 100 single-reads (SR) at an average read depth of 15 million reads/sample. Reads were aligned to the human Refseq (GRCh38) using STAR software (v2.5.2b) (https://github.com/alexdobin/STAR/releases)[58]. Transcript abundance estimation was performed internally in STAR with reference to the GRCh38 annotation using the algorithm of htseq-count. Library size normalization, differential expression and log2 transformation were performed using DESeq2[45]. Gene set and pathway analysis was performed with Gene Set Enrichment Analysis (GSEA)[59]. Ranked datasets contrasting co-cultures versus mono-cultures were tested for enrichment of the BioCarta collection gene sets from the Molecular Signatures Database (MSigDB) ([46] using gene set permutation to test for statistical significance. Heat maps and other visualizations were generated using Partek Genomics software, v.6.6.

### Secretome analyses

For assessment of soluble factors, supernatants of CD4/CD8 co-cultures at 5:1 (E:T) and of CD4 mono-cultures were harvested three days post-infection under single round conditions, either using the replication competent NL4-3_eGFP with 100 nM Darunavir (Janssen Sciences), or the replication defective NL4-3_D2eGFP. Since we kept the total number of cells constant between mono-and co-cultures to avoid variations in media consumption, we were only able to reliably identify the soluble factors that were increased in the 5:1 (E:T CD8/ CD4) supernatants in comparison to the CD4^+^ T cell mono-cultures as a lower yield of a given secreted protein could be an artifact of the lower number of CD4^+^ T cells that are present in the co-cultures in our experimental system. Briefly, at day 3 post-infection the mono- and co-culture cell supernatants were harvested, diluted 2-fold with an inactivation buffer containing 1% 4-Nonylphenyl-polyethylene glycol (Sigma-Aldrich) and 1% protease inhibitor cocktail (Thermo Fisher), and stored at -80°C immediately. A semi-quantitative human cytokine antibody array (C Series 4000, Ray Biotech) detecting 274 proteins was performed to screen soluble factors of mono- and co-cultures per manucturer’s instructions. 1.5 ml of the diluted mono- and co-culture cell supernatants was incubated with each cytokine array at 4°C with gentle shaking, overnight. After development, the chemiluminescent signals were captured using the ChemiDoc XRS+ Imaging system (BioRad), and numerical signal densities were processed with ImageJ software (NIH). The average signal of the pair of duplicate spots representing each cytokine was determined and average background signal from blank was subtracted from each spot. Values that were below background after blank subtraction were set to 0. Values were then normalized using the average internal positive control signals according to manufacturer’s data analysis instructions. This normalization process allows for a consistent comparison of results across multiple arrays. Comparisons between 5:1 (E:T CD8/ CD4) co-cultures and CD4 mono-cultures were carried out using parametric donor-matched pairwise t-test (test of variance) and batch t-test (test of the mean and standard deviation of the set). Normality was assessed using Kolmogorov-Smirnov test, and significance was attributed at p<0.05.

To confirm the T helper 2 (Th2) cytokines IL-4, IL-5, IL-13, and sST2, we used a custom LEGENDplex panel (Biolegend) per manucturer’s instructions. Samples were assayed in duplicate, concentrations determined by flow cytometry, and analysis of the acquired data performed using LEGENDplex Data Analysis Software (version 7.1).

### Statistical analyses

Except for RNA-seq and Cytokine Array, GraphPad Prism 7.0. was used to determine the statistical significance. Comparisons between mono-and co-cultures were determined by paired Wilcoxon matched-pairs signed-rank test, and significance was attributed at P values of <0.05. RNA-seq differential expression analysis and statistical analysis were performed as previously described using the DESeq2 package[45], and significance was attributed at padj <0.05. Cytokine Array statistical analyses were determined using the Scipy v.1.1.0 library implemented in Python v.3.3.1 as previously described using parametric donor-matched pairwise t-test and batch t-test. Normality was assessed using Kolmogorov-Smirnov test, and significance was attributed at p<0.05.

## Supporting information

S1 Fig Short-lived D2eGFP improves observation of inhibitory effect on HIV LTR-driven expression by CD8^+^ T cells in the single cycle infection assay. (**A**) Combined frequencies and MFI of productively infected eGFP^+^ cells are shown for replication competent NL4-3_eGFP virus treated with protease inhibitor Darunavir (indicated by diamonds; n = 6 subjects), and for replication-incompetent Env-defective NL4-3_eGFP complemented in trans with a dual-tropic envelope (indicated by circles; n = 8 subjects). (**B**) Kinetics of fluorescence decay between replication defective NL4-3_eGFP (solid line) and replication defective NL4-3_D2eGFP (dashed line) determined by MFI in matched subjects (n = 2 subjects). (**C**) Comparison of CD8^+^ T mediated suppression activity between replication defective NL4-3_eGFP (solid line) and replication defective NL4-3_D2eGFP (dashed line) determined by frequency and MFI in matched subjects (n = 2 subjects). Comparisons between frequencies and MFI of infection on co-cultures with that of positive control wells (infected CD4^+^ T cells alone) were carried out using Wilcoxon matched-pairs signed rank test.
(TIF)

S2 Fig Comparable regulation of CD4^+^ T cell activation and proliferation by CD8^+^ T cells in the single cycle infection assays. **(A)** HLA-E (MFI) fold increase in stimulated versus resting CD4^+^ T cell subsets (n = 6). **(B-C)** The aggregate data is shown for HLA-DR (MFI), HLA-E (MFI) and CellTrace violet (Fold change in CellTrace violet MFI relative to CD4^+^ T cells alone, followed by a f (x) = 1/x transformation) of uninfected CD4^+^ T cells (mock), non-productively infected and uninfected CD4^+^ T cells (eGFP^-^ /D2eGFP^-^), and productively infected CD4^+^ T cells (eGFP^+^ /D2eGFP^+^). **(B)** Experiments conducted with replication competent NL4-3_eGFP virus treated with protease inhibitor Darunavir are indicated by diamonds (n = 6 subjects), and with replication-incompetent Env-defective NL4-3_eGFP complemented in trans with a dual-tropic envelope are indicated by circles (n = 8 subjects). (**C**) Infection with Env-defective NL4-3_D2eGFP virus. CD4 mono-culture wells (black), CD4/CD8 at 1:1 (blue) and 5:1 (red) E:T ratios from each subject (n = 7 subjects). Comparisons between frequencies and MFI of infection on mono- and co-cultures were carried out using Wilcoxon matched-pairs signed rank test.
(TIF)

S3 Fig Gating Strategy for sorted eGFP^-^ and eGFP^+^ CD4^+^ T cell subsets. FACS-sorting was performed three days post-infection using the replication competent NL4-3_eGFP under single cycle condition with 100 nM Darunavir. The uninfected (mock) wells were used as negative controls to draw a gate for the HIV-infected (eGFP) wells, which were subsequently sorted as productively infected eGFP^+^CD4^+^ T cell population derived from live CD3^+^Vio^+^Red^-^CD8^-^eGFP^+^ and non-productively infected as well as uninfected eGFP^-^CD4^+^ T cell population derived from live CD3^+^Vio^+^Red^-^CD8^-^GFP^-^ (See Methods). **(A)** Representative sorted cells derived from CD4 mono-cultures and (**B**) from CD4/CD8 co-cultures.
(TIF)

S4 Fig GSEA reveals downregulation of multiple genes associated with cell death, proliferation, Th differentiation and inflammation by CD8^+^ T cells. Data shown are the leading-edge/core enriched genes that account for the gene set’s enrichment signal depicted in **Fig 5C** (GSEA barplots), for Fas-signaling pathway (cell apoptosis), G2/M Checkpoint pathway (cell proliferation and DNA repair), Th1/Th2 and Inflammatory pathways. The leading-edge selected for enrichment testing were obtained from the MSigDB database BioCarta collection and are denoted at the right of each panel. Genes are ordered from top to bottom by increasing normalized enrichment score (NES) of the eGFP^-^ co-cultured versus mono-cultured samples. Values are the log2-transformed difference between CD4/CD8 co-cultures and CD4 mono-cultures for each individual subject (n = 8 subjects) and distinct viral production (eGFP^+^ and eGFP^-^). Values are log2-transformed and "mean baseline normalized” to show the relative difference in expression with respect to the mean of all samples. The range of differential expression shown is the same (-0.3 to 0.3 log2) for all but the Inflammatory Pathway which has a range from (-0.1 to 0.1 log2). The color scale denotes the maximum and minimum on a log2 scale.
(TIF)

S5 Fig Purity of CD8^+^ T cells enriched from PBMC. CD8^+^ T cells from HIV-negative healthy subjects were enriched by negative selection as described in Methods, and purity was assessed by flow cytometry. Cells were initially gated on singlets (FSC-H versus FSC-A), on the basis of light scatter (SSC-A versus FSC-A), followed by a negative staining for Live/Dead Aqua. CD8^+^ T cell enrichment is demonstrated on a CD3 versus CD8 plot to exclude CD8^+^ non-T cells such as DC or NK populations.
(TIF)

S6 Fig Th2 cytokines alone or in combination do not suppress HIV expression in infected CD4^+^ T cells cultured *in vitro*. Solid and dashed lines represent the mean fluorescence intensity (MFI) values of D2eGFP^+^ cells of 4 distinct subjects (n = 4). Gray area indicates HIV suppression mediated by autologous CD8^+^ T cells of the same subjects (n = 4).
(TIF)

S7 Fig Blockage of Th2 cytokines does not rescue HIV expression in CD4/CD8 co-cultures. CD4/CD8 T cell co-cultures were either treated with anti- (IL-4, -5, -13, and -33R) blocking mAbs (10 μg/ml), or with the correspondent isotype controls (10 μg/ml) after infection. CD4/CD8 at 1:1 (blue) and 5:1 (red) effector-to-target cell (E:T) ratios, from each subject (n = 4 subjects).
(TIF)

S8 Fig Venn diagrams demonstrating differentially expressed genes (DEGs) in eGFP- and eGFP+CD4+ T subsets in response to CD8^+^ T cells (DESeq2: padj <0.05 and fold change threshold >1.5 (0.585 log) or <0.67 (-0.585 log). Intersection shows DEGs in common between eGFP- and eGFP+CD4^+^ T subsets in response to CD8^+^ T cells.
(TIF)

S1 Table Differentially expressed soluble factors between 1CD4/5CD8 and CD4 T cells. Abbreviations are as follows: CSF1R/ M-CSFR, macrophage colony-stimulating factor receptor; G-CSF, granulocyte colony- stimulating factor; GITR, glucocorticoid-induced TNF family-related receptor; GITR Ligand, glucocorticoid-induced tumor necrosis factor receptor-related protein ligand; PDGFR alpha, receptor for platelet-derived growth factor alpha; TIE-1, tyrosine kinase with immunoglobulin like and EGF like domains 1. ^a^ Solube factors are ranked on the basis of fold-increase between 1CD4/5CD8 co-culture and CD4 mono-culture (n = 6 subjects). Significance was attributed at p<0.05 using paired t-test. Asterisks (*) indicate statistical significance (p<0.05) using batch t test.
(DOCX)
