# Supplementary figures and images for: Innate, non-cytolytic CD8+ T cell-mediated suppression of HIV replication by MHC-independent inhibition of virus transcription
Source: PLoS Pathog. 2020 Sep 17;16(9):e1008821. doi: 10.1371/journal.ppat.1008821 (PMC7523993; doi:10.1371/journal.ppat.1008821)

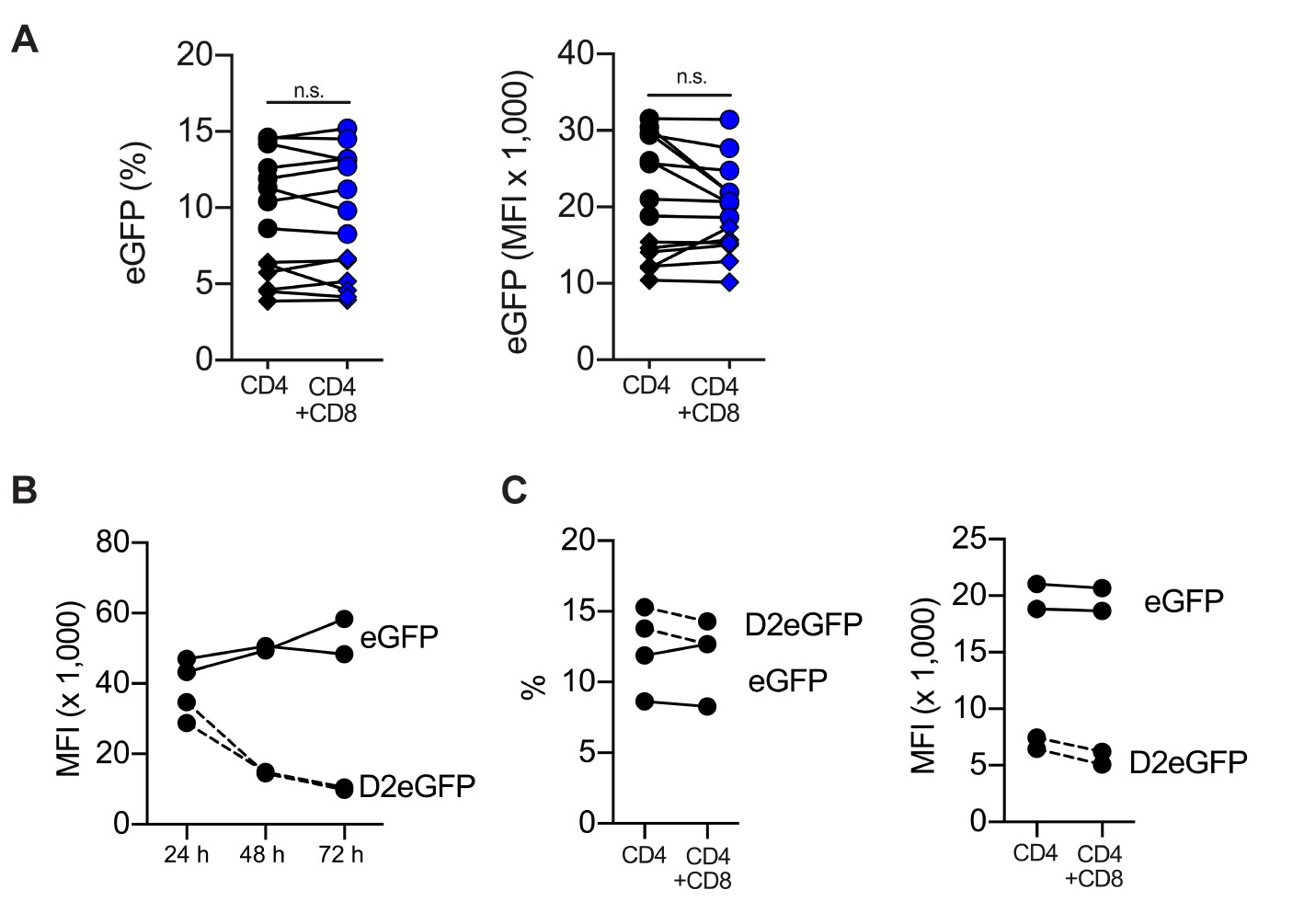

Supplement: S1 Fig — (A) Combined frequencies and MFI of productively infected eGFP+ cells are shown for replication competent NL4-3_eGFP virus treated with protease inhibitor Darunavir (indicated by diamonds; n = 6 subjects), and for replication-incompetent Env-defective NL4-3_eGFP complemented in trans with a dual-tropic envelope (indicated by circles; n = 8 subjects). (B) Kinetics of fluorescence decay between replication defective NL4-3_eGFP (solid line) and replication defective NL4-3_D2eGFP (dashed line) determined by MFI in matched subjects (n = 2 subjects). (C) Comparison of CD8+ T mediated suppression activity between replication defective NL4-3_eGFP (solid line) and replication defective NL4-3_D2eGFP (dashed line) determined by frequency and MFI in matched subjects (n = 2 subjects). Comparisons between frequencies and MFI of infection on co-cultures with that of positive control wells (infected CD4+ T cells alone) were carried out using Wilcoxon matched-pairs signed rank test. (TIF) [file ppat.1008821.s001.tif]

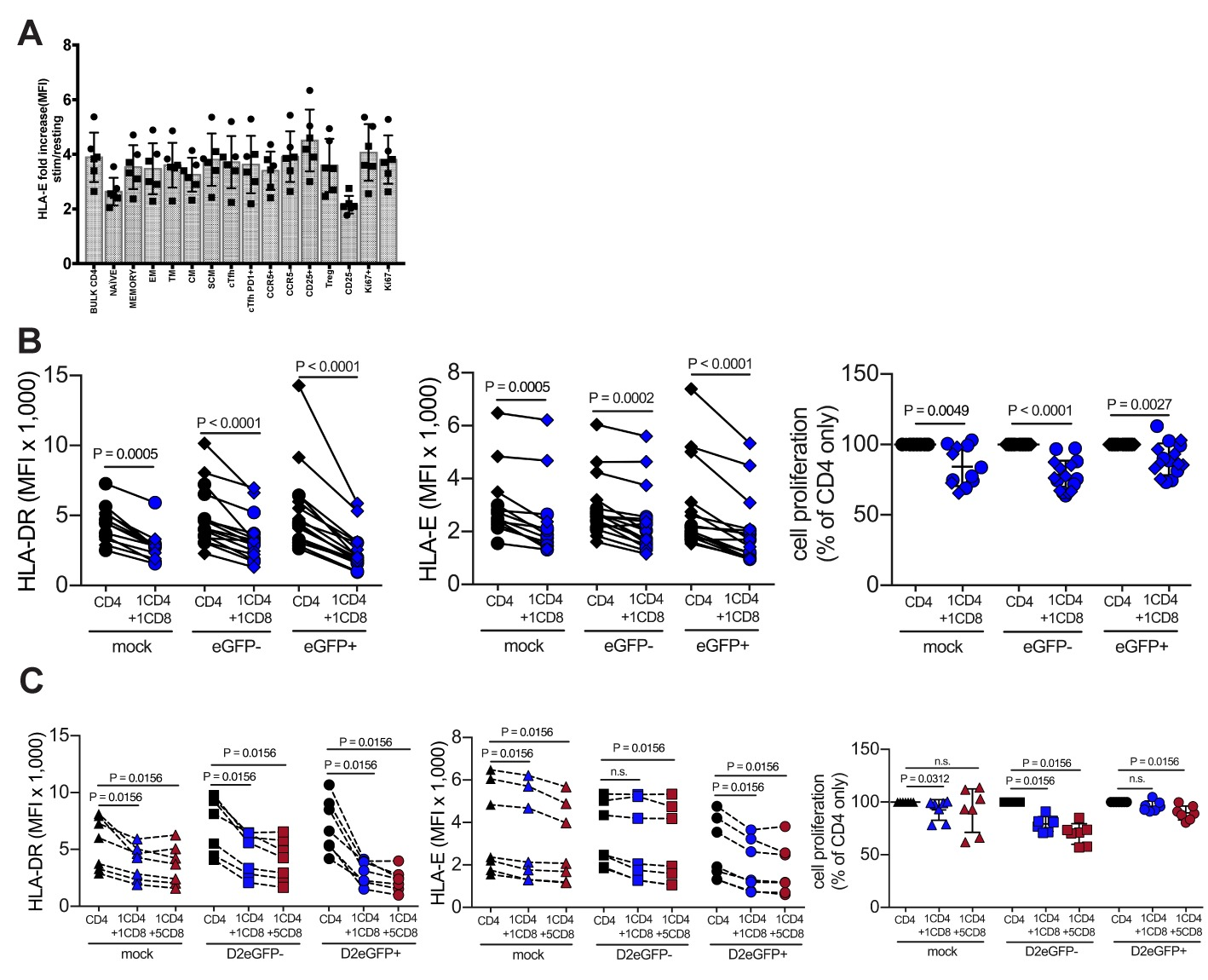

Supplement: S2 Fig — (A) HLA-E (MFI) fold increase in stimulated versus resting CD4+ T cell subsets (n = 6). (B-C) The aggregate data is shown for HLA-DR (MFI), HLA-E (MFI) and CellTrace violet (Fold change in CellTrace violet MFI relative to CD4+ T cells alone, followed by a f (x) = 1/x transformation) of uninfected CD4+ T cells (mock), non-productively infected and uninfected CD4+ T cells (eGFP- /D2eGFP-), and productively infected CD4+ T cells (eGFP+ /D2eGFP+). (B) Experiments conducted with replication competent NL4-3_eGFP virus treated with protease inhibitor Darunavir are indicated by diamonds (n = 6 subjects), and with replication-incompetent Env-defective NL4-3_eGFP complemented in trans with a dual-tropic envelope are indicated by circles (n = 8 subjects). (C) Infection with Env-defective NL4-3_D2eGFP virus. CD4 mono-culture wells (black), CD4/CD8 at 1:1 (blue) and 5:1 (red) E:T ratios from each subject (n = 7 subjects). Comparisons between frequencies and MFI of infection on mono- and co-cultures were carried out using Wilcoxon matched-pairs signed rank test. (TIF) [file ppat.1008821.s002.tif]

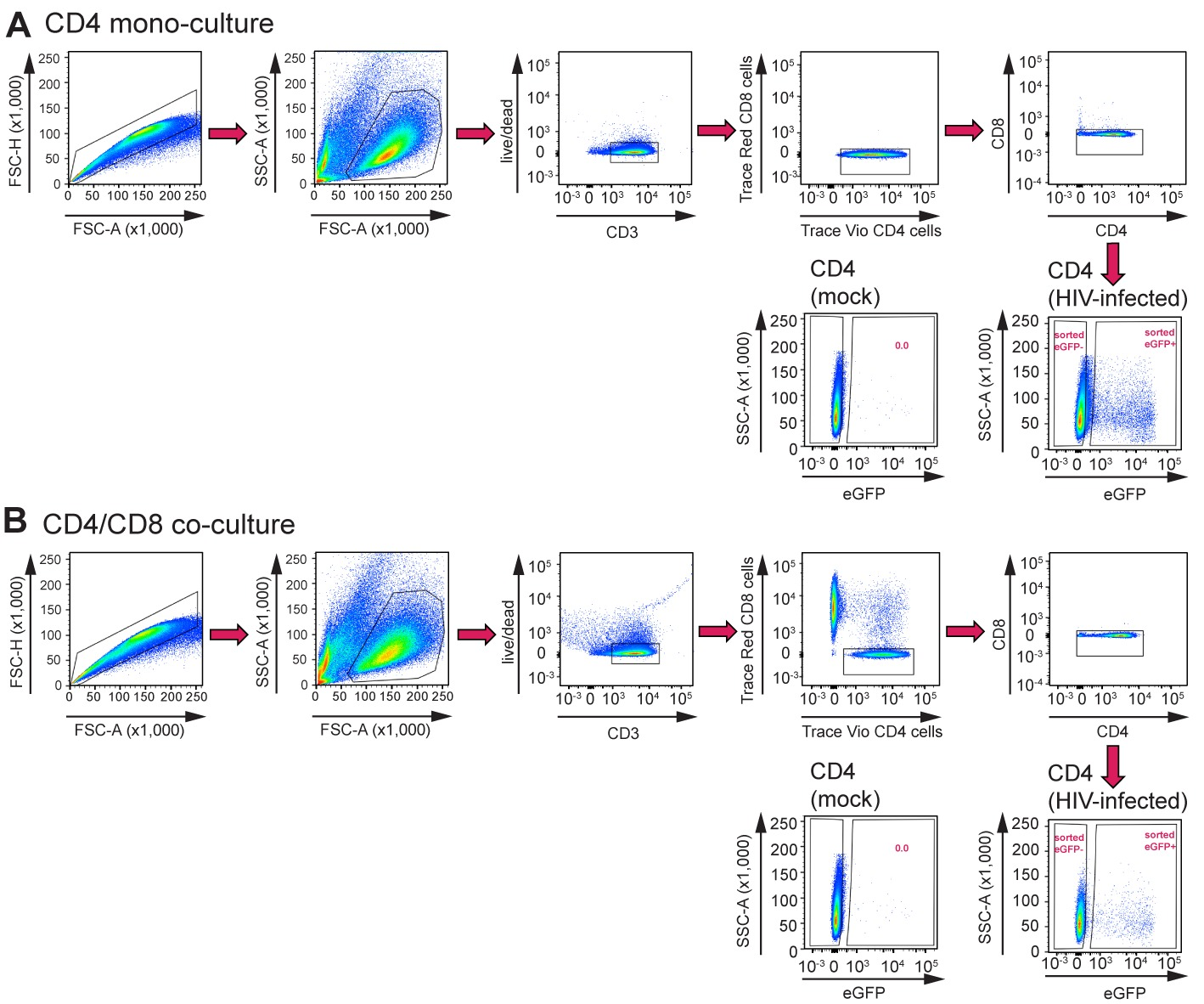

Supplement: S3 Fig — FACS-sorting was performed three days post-infection using the replication competent NL4-3_eGFP under single cycle condition with 100 nM Darunavir. The uninfected (mock) wells were used as negative controls to draw a gate for the HIV-infected (eGFP) wells, which were subsequently sorted as productively infected eGFP+CD4+ T cell population derived from live CD3+Vio+Red-CD8-eGFP+ and non-productively infected as well as uninfected eGFP-CD4+ T cell population derived from live CD3+Vio+Red-CD8-GFP- (See Methods). (A) Representative sorted cells derived from CD4 mono-cultures and (B) from CD4/CD8 co-cultures. (TIF) [file ppat.1008821.s003.tif]

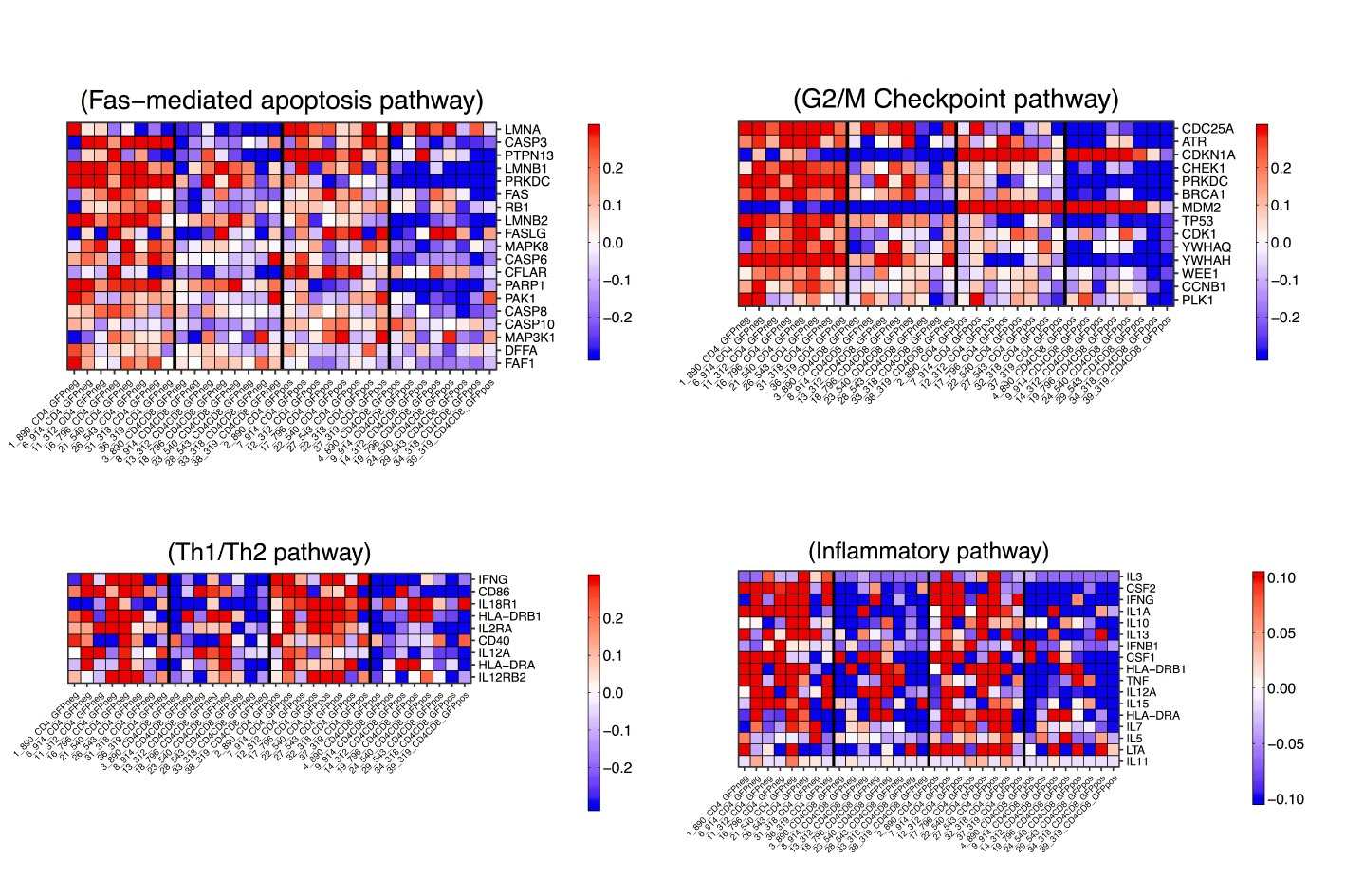

Supplement: S4 Fig — Data shown are the leading-edge/core enriched genes that account for the gene set’s enrichment signal depicted in Fig 5C (GSEA barplots), for Fas-signaling pathway (cell apoptosis), G2/M Checkpoint pathway (cell proliferation and DNA repair), Th1/Th2 and Inflammatory pathways. The leading-edge selected for enrichment testing were obtained from the MSigDB database BioCarta collection and are denoted at the right of each panel. Genes are ordered from top to bottom by increasing normalized enrichment score (NES) of the eGFP- co-cultured versus mono-cultured samples. Values are the log2-transformed difference between CD4/CD8 co-cultures and CD4 mono-cultures for each individual subject (n = 8 subjects) and distinct viral production (eGFP+ and eGFP-). Values are log2-transformed and "mean baseline normalized” to show the relative difference in expression with respect to the mean of all samples. The range of differential expression shown is the same (-0.3 to 0.3 log2) for all but the Inflammatory Pathway which has a range from (-0.1 to 0.1 log2). The color scale denotes the maximum and minimum on a log2 scale. (TIF) [file ppat.1008821.s004.tif]

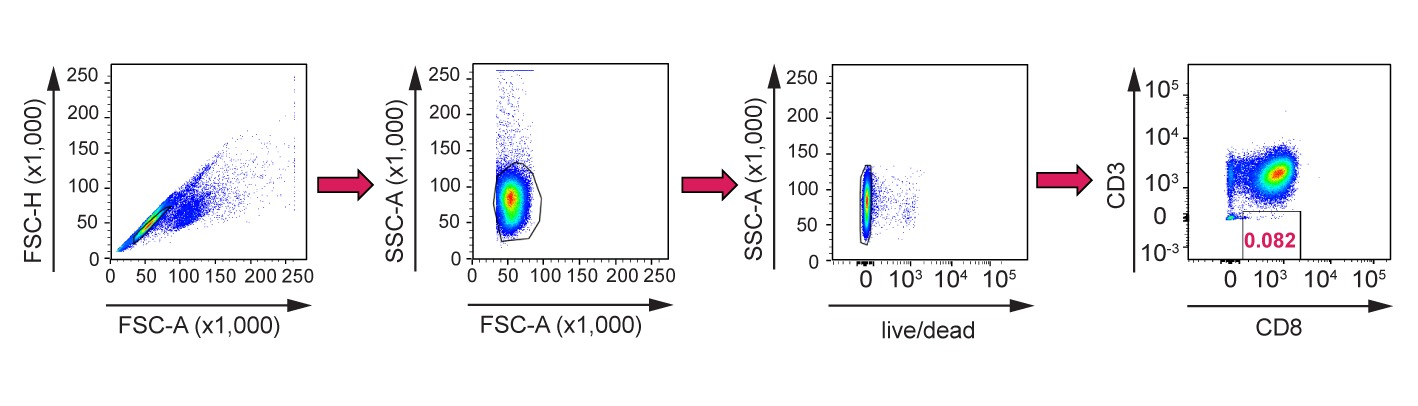

Supplement: S5 Fig — CD8+ T cells from HIV-negative healthy subjects were enriched by negative selection as described in Methods, and purity was assessed by flow cytometry. Cells were initially gated on singlets (FSC-H versus FSC-A), on the basis of light scatter (SSC-A versus FSC-A), followed by a negative staining for Live/Dead Aqua. CD8+ T cell enrichment is demonstrated on a CD3 versus CD8 plot to exclude CD8+ non-T cells such as DC or NK populations. (TIF) [file ppat.1008821.s005.tif]

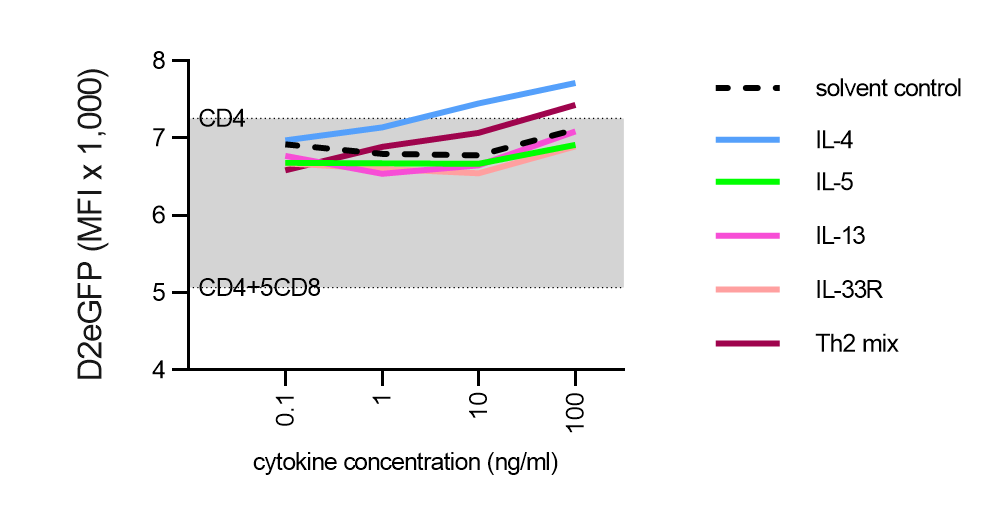

Supplement: S6 Fig — Solid and dashed lines represent the mean fluorescence intensity (MFI) values of D2eGFP+ cells of 4 distinct subjects (n = 4). Gray area indicates HIV suppression mediated by autologous CD8+ T cells of the same subjects (n = 4). (TIF) [file ppat.1008821.s006.tif]

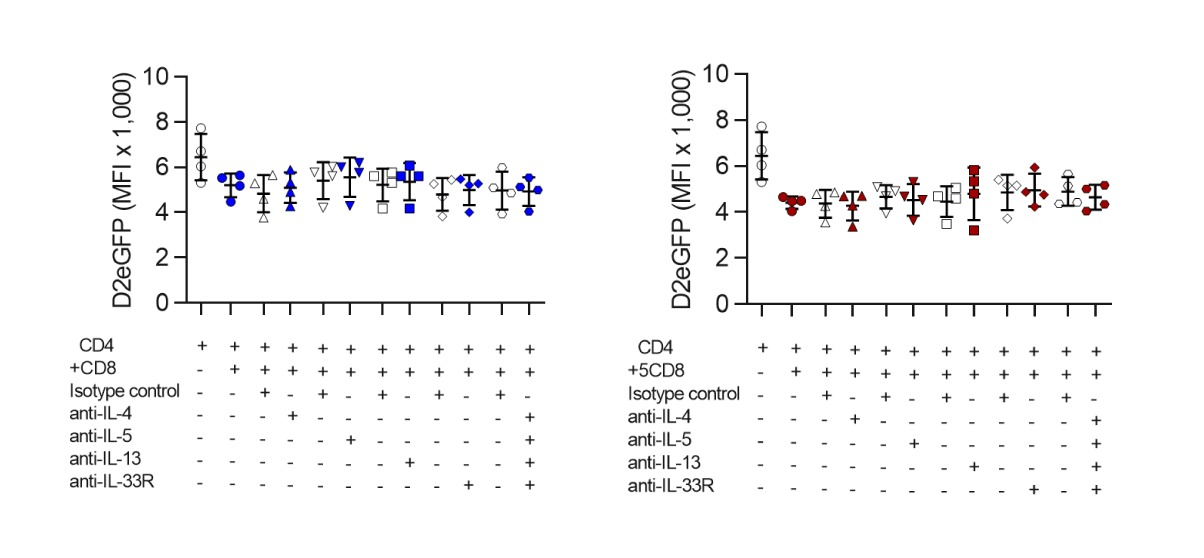

Supplement: S7 Fig — CD4/CD8 T cell co-cultures were either treated with anti- (IL-4, -5, -13, and -33R) blocking mAbs (10 μg/ml), or with the correspondent isotype controls (10 μg/ml) after infection. CD4/CD8 at 1:1 (blue) and 5:1 (red) effector-to-target cell (E:T) ratios, from each subject (n = 4 subjects). (TIF) [file ppat.1008821.s007.tif]

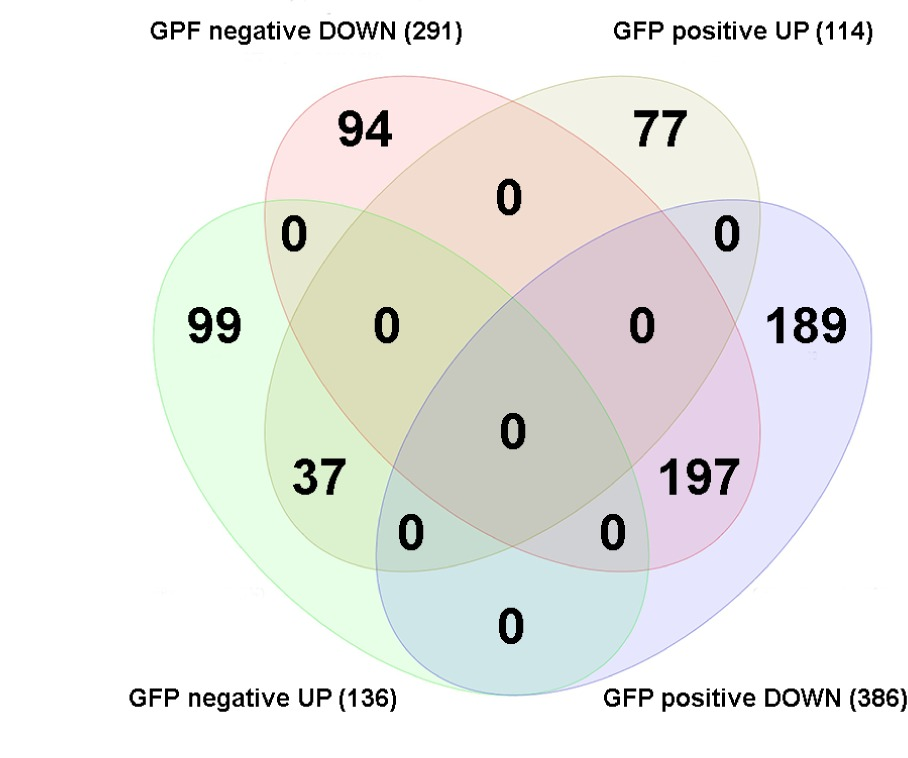

Supplement: S8 Fig — Intersection shows DEGs in common between eGFP- and eGFP+CD4+ T subsets in response to CD8+ T cells. (TIF) [file ppat.1008821.s008.tif]
