# Supplementary material for: Innate, non-cytolytic CD8+ T cell-mediated suppression of HIV replication by MHC-independent inhibition of virus transcription
Source: PLoS Pathog. 2020 Sep 17;16(9):e1008821. doi: 10.1371/journal.ppat.1008821 (PMC7523993; doi:10.1371/journal.ppat.1008821)
Supplement: S1 Table — Abbreviations are as follows: CSF1R/ M-CSFR, macrophage colony-stimulating factor receptor; G-CSF, granulocyte colony- stimulating factor; GITR, glucocorticoid-induced TNF family-related receptor; GITR Ligand, glucocorticoid-induced tumor necrosis factor receptor-related protein ligand; PDGFR alpha, receptor for platelet-derived growth factor alpha; TIE-1, tyrosine kinase with immunoglobulin like and EGF like domains 1. a Solube factors are ranked on the basis of fold-increase between 1CD4/5CD8 co-culture and CD4 mono-culture (n = 6 subjects). Significance was attributed at p<0.05 using paired t-test. Asterisks (*) indicate statistical significance (p<0.05) using batch t test. (DOCX) [file ppat.1008821.s009.docx]

| S1 Table. Differentially expressed soluble factors between 1CD4/5CD8 and CD4 T cells | | | | |
| --- | --- | --- | --- | --- |
| Soluble factor^a^ | **Description** | **n fold-increase** | **P-value** |  |
| IL-13 R alpha 2 | Th2-type cytokine receptor | 180 | 0.005 |  |
| MMP-9 | Matrix metalloproteinase-9 | 85.8 | 0.02 |  |
| IL-9 | Th2-type cytokine | 59.5 | 0.0009 |  |
| MMP-3 | Matrix metalloproteinase-3 | 31.2 | 0.05 |  |
| SDF-1 beta | CXCR4 ligand | 22.3 | 0.04 |  |
| IL-18 R beta (AcPL) | Th1-type cytokine receptor | 19.8 | 0.008 |  |
| CSF1R/M-CSFR | Member of the CSF1/PDGF receptor family of tyrosine-protein kinases | 18.1 | 0.02 |  |
| PDGFR alpha | Member of the CSF1/PDGF receptor family of tyrosine-protein kinases | 17.3 | 0.03 |  |
| IL-1 R2 | IL-1β Inhibitor | 8.9 | 0.05* |  |
| TIE-1 | Anti-apoptotic (via PI3K-AKT pathway) | 7.5 | 0.05* |  |
| G-CSF | Modulates T-cell function toward a type-2 response in mice/ T-regulatory in humans | 4.5 | 0.03* |  |
| IL-5 | Th2-type cytokine | 3.4 | 0.04* |  |
| IL-1 R4 (ST2), IL-33 R | Th2-type cytokine receptor | 3.1 | 0.05* |  |
| TRAIL-R4 | Anti-apoptotic (protects against TRAIL-Mediated Apoptosis) | 3.1 | 0.04* |  |
| ICAM-1 | Adhesion molecule | 2.3 | 0.03 |  |
| GITR | Anti-inflammatory and anti-apoptotic | 1.94 | 0.02 |  |
| GITR Ligand | Anti-inflammatory and anti-apoptotic | 1.72 | 0.01 |  |
| IL-13 | Th2-type cytokine | 1.6 | 0.04 |  |
| Abbreviations are as follows: CSF1R/ M-CSFR, macrophage colony-stimulating factor receptor; G-CSF, granulocyte colony- stimulating factor; GITR, glucocorticoid-induced TNF family-related receptor; GITR Ligand, glucocorticoid-induced tumor necrosis factor receptor-related protein ligand; PDGFR alpha, receptor for platelet-derived growth factor alpha; TIE-1, tyrosine kinase with immunoglobulin like and EGF like domains 1.  ^a^ Solube factors are ranked on the basis of fold-increase between 1CD4/5CD8 co-culture and CD4 mono-culture (n = 6 subjects). Significance was attributed at p<0.05 using paired t-test. Asterisks (*) indicate statistical significance (p<0.05) using batch t test. | | | | |
